# STAG2 inactivation reprograms glutamine metabolism of BRAF-mutant thyroid cancer cells

**DOI:** 10.1038/s41419-023-05981-z

**Published:** 2023-07-21

**Authors:** Xinru Li, Yan Liu, Juan Liu, Wei Qiang, Jingjing Ma, Jingyi Xie, Pu Chen, Yubo Wang, Peng Hou, Meiju Ji

**Affiliations:** 1 Key Laboratory for Tumor Precision Medicine of Shaanxi Province and Department of Endocrinology, The First Affiliated Hospital of Xi’an Jiaotong University, Xi’an, 710061 PR China; 2 Department of Nuclear Medicine, The First Affiliated Hospital of Xi’an Jiaotong University, Xi’an, 710061 PR China; 3 Department of Endocrinology, The First Affiliated Hospital of Xi’an Jiaotong University, Xi’an, 710061 PR China; 4 Center for Translational Medicine, The First Affiliated Hospital of Xi’an Jiaotong University, Xi’an, 710061 PR China

**Keywords:** Thyroid cancer, Cancer metabolism

## Abstract

STAG2, an important subunit in cohesion complex, is involved in the segregation of chromosomes during the late mitosis and the formation of sister chromatids. Mutational inactivation of *STAG2* is a major cause of the resistance of BRAF-mutant melanomas to BRAF/MEK inhibitors. In the present study, we found that STAG2 was frequently down-regulated in thyroid cancers compared with control subjects. By a series of in vitro and in vivo studies, we demonstrated that STAG2 knockdown virtually had no effect on malignant phenotypes of BRAF-mutant thyroid cancer cells such as cell proliferation, colony formation and tumorigenic ability in nude mice compared with the control. In addition, unlike melanoma, STAG2 knockdown also did not affect the sensitivity of these cells to MEK inhibitor. However, we surprisingly found that STAG2-knockdown cells exhibited more sensitive to glutamine deprivation or glutaminase inhibitor BPTES compared with control cells. Mechanistically, knocking down STAG2 in BRAF-mutant thyroid cancer cells decreases the protein stability of c-Myc via the ERK/AKT/GSK3β feedback pathway, thereby impairing glutamine metabolism of thyroid cancer cells by down-regulating its downstream targets such as SCL1A5, GLS and GLS2. Our data, taken together, demonstrate that STAG2 inactivation reprograms glutamine metabolism of BRAF-mutant thyroid cancer cells, thereby improving their cellular response to glutaminase inhibitor. This study will provide a potential therapeutic strategy for BRAF-mutant thyroid cancers.

## Introduction

In recent years, the incidence of thyroid cancer has dramatically increased in the world, including China [1–3]. Although the majority of thyroid cancers are differentiated thyroid cancers (DTCs), which usually have a good prognosis and can be almost cured by conventional therapies [4, 5], some patients still develop more aggressive diseases such as metastatic DTCs, radioactive iodine-resistance (RAIR) DTC and even anaplastic thyroid cancers (ATCs), with no effective therapies available. Thus, there is pressing need to develop additional therapeutic options for these kinds of diseases.

As the most common genetic alteration in thyroid cancers, BRAF^V600E^ has been reported to be associated with aggressive clinical behaviors such as metastasis-associated mortality risk, RAIR-DTC and even one of the predominant drivers in ATCs [6–10]. Targeting BRAF^V600E^ has thus become a major therapeutic target [11]. However, unlike melanoma, the clinical benefit of inhibitors against this mutation in thyroid cancers is often limited by short-lived responses and acquired resistance via heterogeneous feedback mechanisms [12]. Stromal antigen 2 (STAG2) as a crucial subunit of the cohesin complex is involved in the separation of sister chromatids during late mitosis and the formation of sister chromatids [13, 14]. There is evidence supporting that mutational inactivation of *STAG2* is a major cause of the resistance of BRAF-mutant melanomas to BRAF/MEK inhibitors [15]. However, its biological role in thyroid cancer and its effect on the cellular response of BRAF-mutant thyroid cancer cells to BRAF/MEK inhibitors remains totally unclear.

In this study, we observed that although STAG2 was down-regulated in thyroid cancers and knocking down STAG2 in BRAF-mutant thyroid cancer cells caused a conspicuous up-regulation of phosphorylated ERK (p-ERK), it virtually had no effect on malignant phenotypes of these cells and their response to MEK inhibitor. However, our data demonstrated that STAG2 inactivation significantly reduced the ability of these cells to uptake/utilize glutamine via the ERK/AKT/GSK3β/c-Myc feedback axis. As a result, it will cause these cells to be more sensitive to glutaminase inhibitor.

## Results

### STAG2 knockdown has no effect on the proliferation and colony formation of BRAF-mutant thyroid cancer cells

**Fig. 1:**
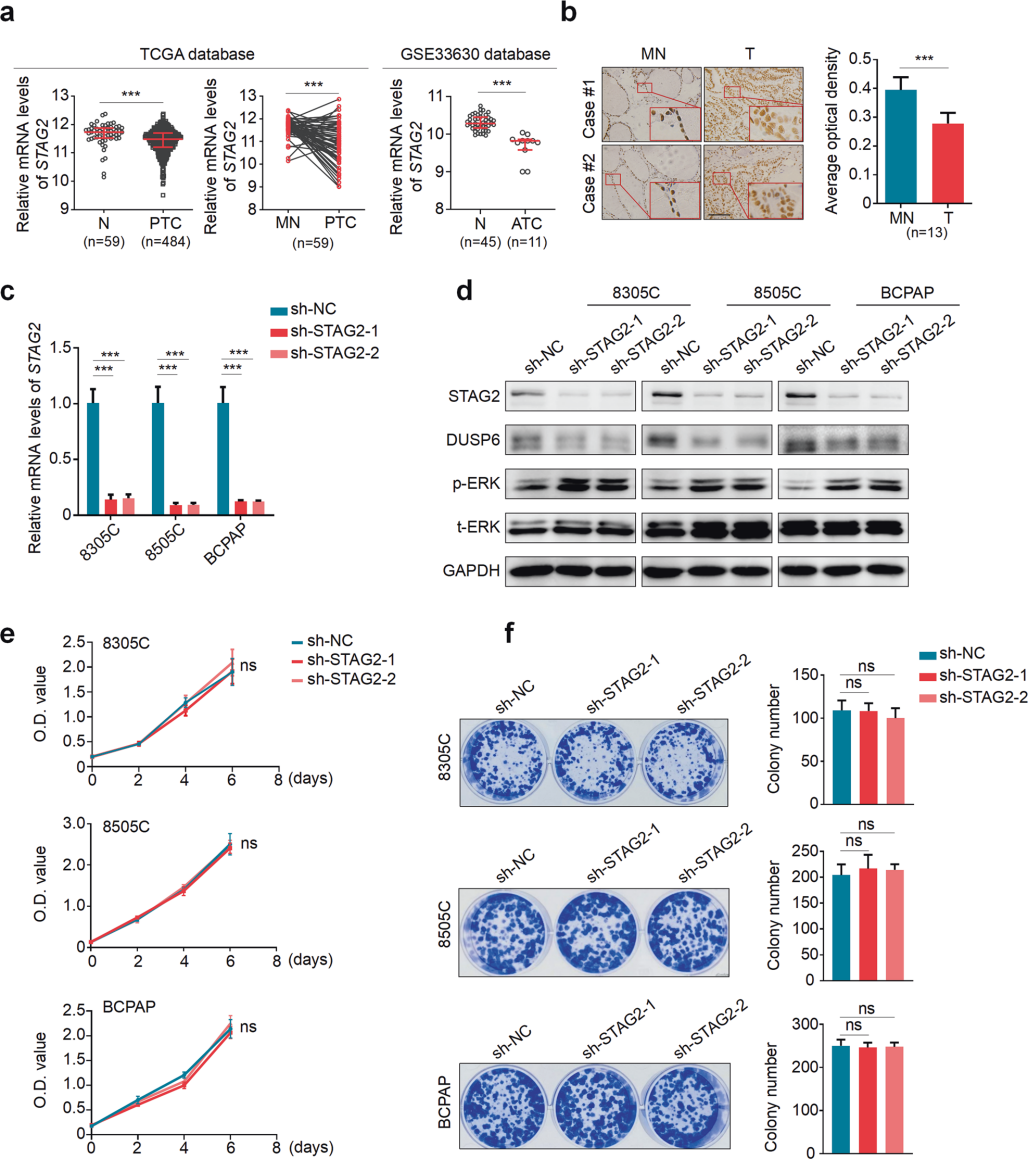
The effect of STAG2 knockdown on the proliferation and colony formation of BRAF-mutant thyroid cancer cells. **a** The expression of STAG2 in papillary thyroid cancers (PTC) compared with non-cancerous (N) or matched non-cancerous tissues (MN) from TCGA database and in ATCs compared with normal thyroid tissues (N) from GEO database (GSE33630). **b** Comparison of STAG2 expression between PTCs (*n* = 13) and their matched non-cancerous tissues (MN) by immunohistochemical (IHC) method. Left panels show representative images of IHC. Scale bar, 200 μm. Average optical density value was automatic calculated using ImageJ software. **c** Knockdown efficiency of STAG2 was validated by qRT-PCR. β-actin was used as an internal reference. **d** Western blot analysis of STAG2, DUSP6, phosphorylated ERK (p-ERK) and total ERK (t-ERK) in STAG2-knockdown 8305C, 8505C and BCPAP cells and their control cells. GAPDH was used as a loading control. **e** Comparison of cell proliferation between STAG2-knockdown 8305C, 8505C and BCPAP cells and their control cells by MTT assay. **f** Comparison of colony formation ability between STAG2-knockdown 8305C, 8505C and BCPAP cells and their control cells assays. Left panels show representative images of colony formation. Quantitative analysis of colony numbers is presented in the right panels. Data are presented as mean ± SD of values from three different experiments. ns no significance; **P < 0.01; ***P < 0.001.

We first analyzed *STAG2* expression in papillary thyroid cancers (PTCs) from The Cancer Genome Atlas (TCGA) database and Gene Expression Omnibus (GEO) database (GSE33630). The results showed that *STAG2* expression was significantly down-regulated in PTCs and ATCs in comparison with control subjects (Fig. 1a). We also evaluated its expression in 13 PTCs and their matched non-cancerous tissues by immunohistochemistry (IHC) assay. The results further supported the above conclusion (Fig. 1b).

Next, we evaluated the effect of STAG2 downregulation on cell proliferation and colony formation in 8305C, 8505C and BCPAP cells. The results showed that knocking down STAG2 in these cells clearly decreased the expression of dual specificity phosphatase 6 (DUSP6) and elevated the level of p-ERK compared with the control (Fig. 1c, d; Supplementary Fig. 1); however, it almost did not affect the proliferation and colony formation of these cells (Fig. 1e, f). Meanwhile, the similar results were found in melanoma cell line A375 (Supplementary Fig. 2), which was consistent with a previous study [15].

### STAG2 knockdown has no effect on the response of BRAF-mutant thyroid cancer cells to MEK inhibitor

**Fig. 2:**
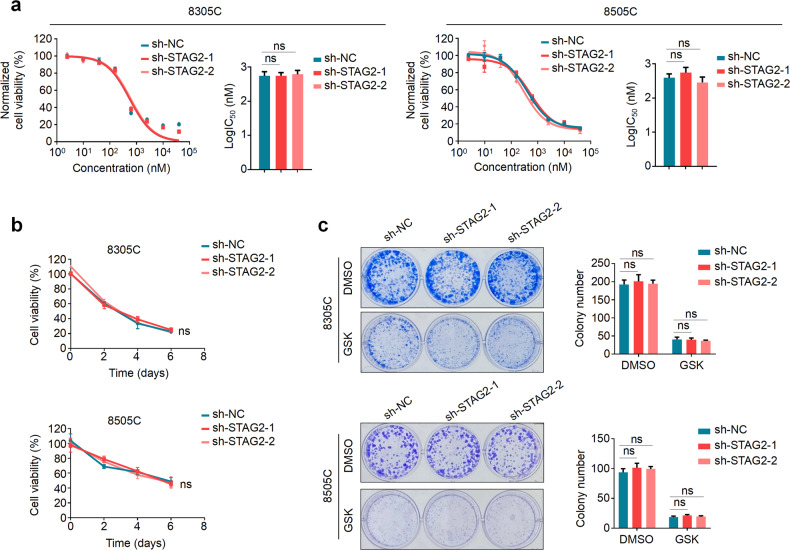
The effect of STAG2 knockdown on response of BRAF-mutant thyroid cancer cells to MEK inhibitor. **a** STAG2-knockdown 8305C and 8505C and their control cells were treated with different concentrations of MEK inhibitor GSK1120212 (GSK) for 72 h, and their effect on cell proliferation was assessed by MTT assay. The Reed-Muench method was used to calculate Log IC_50_ values. **b** The time-dependent cellular response was assessed by MTT assay when the above cells were treated with 500 nM GSK for indicated time points. **c** STAG2-knockdown 8305C and 8505C and their control cells were treated with the 500 nM GSK or equivalent DMSO for 7–10 days, and then stained with crystal violet. The left panels show representative images of colony formation. Quantitative analysis of colony numbers is shown in the right panels. Data are presented as mean ± SD of values from three different experiments. ns no significance.

Considering that knocking down STAG2 in BRAF-mutant melanoma cells significantly decreased their sensitivity to BRAF/MEK inhibitors [15], we thus attempted to determine whether STAG2 knockdown affected the response of BRAF-mutant thyroid cancer cells to BRAF/MEK inhibitors. In addition, given that BRAF-mutant thyroid cancer cells usually showed intrinsic resistance to BRAF kinase inhibitors due to the feedback activation of epidermal growth factor receptor 3 (EGFR3/HER3) [12], thus, we only tested the response of these cells to MEK inhibitor GSK1120212 in the present study. The results showed that knocking down STAG2 in 8305C, 8505C and BCPAP cells did not affect their cellular response to GSK1120212 by a series of dose- and time-dependent experiments (Fig. 2a, b; Supplementary Fig. 3a). Meanwhile, colony formation assay further supported the above conclusion (Fig. 2c; Supplementary Fig. 3b). In a sharp contrast, we knocked down STAG2 in A375 cells and found that these cells showed resistance to BRAF kinase inhibitor PLX4032 or/and GSK1120212 in a dose- and time-dependent manner (Supplementary Fig. 4a, b), which was consistent with a previous study [15]. Collectively, these results indicate that, although STAG2 knockdown activates ERK signaling in both BRAF-mutant thyroid cancer cells and melanoma cells, it causes distinct cellular response to MEK inhibitor.

### STAG2 inactivation increases cellular response of thyroid cancer cells to glutamine deprivation and glutaminase inhibitor

**Fig. 3:**
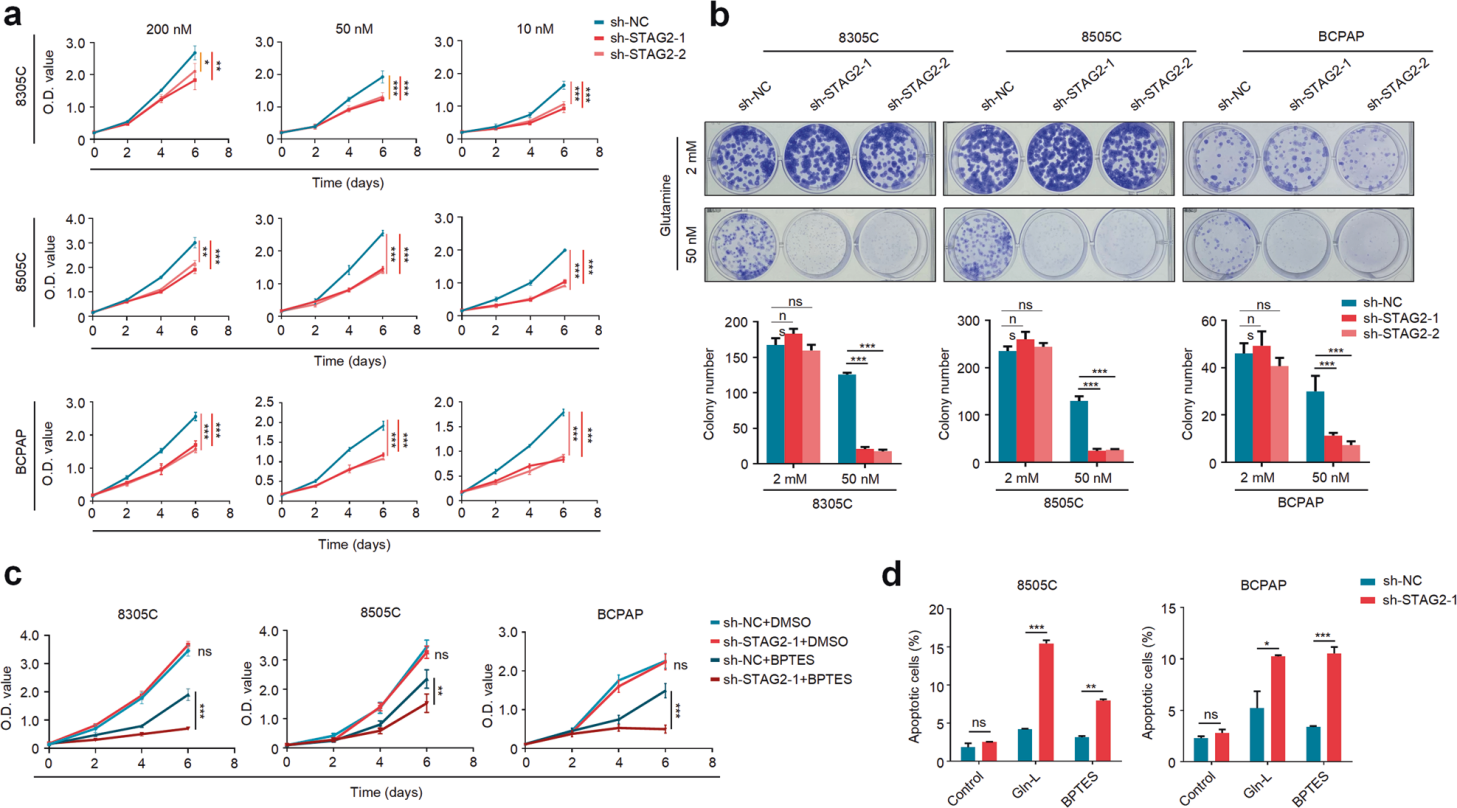
STAG2 knockdown renders BRAF-mutant thyroid cancer cells more sensitive to glutamine deprivation or glutaminase inhibition. **a** STAG2-knockdown 8305C, 8505C and BCPAP and their control cells were cultured in the RPMI 1640 medium containing low concentrations (200 nM, 50 nM and 10 nM) of glutamine, and their proliferation ability was then assessed by MTT assay. **b** Colony formation ability was assessed after the above cells were cultured in RPMI 1640 medium containing 2 mM (standard) or 50 nM of glutamine for appropriate time. The upper panels show representative images and quantitative analysis of colony numbers is shown in the lower panels. **c** STAG2-knockdown thyroid cancer cells and their control cells were cultured in the RPMI 1640 medium containing 2 mM of glutamine or were treated with 8 μM BPTES or equivalent DMSO, and their effect on cell proliferation was then assessed by MTT assay. **d** STAG2-knockdown 8505C and BCPAP cells and their control cells were cultured in the medium containing 2 mM glutamine (Control) or 50 nM of glutamine (Gln-L) or were treated with 8 μM BPTES in the normal medium for 48 h, and apoptotic cells were then detected by flow cytometry using Annexin V-FITC Detection Kit. The percentage of apoptotic cells calculated as the sum of the early and late apoptosis was presented. Data are presented as mean ± SD from three different experiments. ns no significance; *P < 0.05; **P < 0.01; ***P < 0.001.

Glucose and glutamine as fundamental energy substrates are required for cell division, and cancer cells need to increase glucose and glutamine flux to provide their energy needs [16, 17]. Thus, we attempted to determine whether STAG2 inactivation affected glucose or glutamine metabolism of BRAF-mutant thyroid cancer cells. The results showed that glucose deprivation did not cause difference in cell proliferation between STAG2-knockdown 8305C, 8505C and BCPAP cells with control ones (Supplementary Fig. 5). However, we strikingly found that knocking down STAG2 in 8305C, 8505C and BCPAP cells significantly suppressed cell proliferation compared with the control when these cells were cultured in the medium containing relatively low concentrations of glutamine (200 nM, 50 nM and 10 nM) (Fig. 3a). As supported, we also observed a similar finding in colony formation assay (Fig. 3b). Unlike thyroid cancer cells, STAG2 knockdown in A375 cells had no effect on cell proliferation when deprived of glutamine (Supplementary Fig. 6), further supporting that STAG2 may exert distinct roles in different types of cancer.

We next treated thyroid cancer cells with glutaminase inhibitor BPTES to evaluate its effect on cell proliferation. The results showed that STAG2 knockdown caused a conspicuous growth inhibition compared with the control when treated with BPTES (Fig. 3c). Besides, considering that glutamine deficiency/deprivation can induce apoptosis by triggering intrinsic apoptotic pathway in cancer cells [18], we determined the effect of glutamine deprivation or BPTES on cell apoptosis. The results showed that the percentages of apoptotic cells were significantly increased in STAG2-knockdown cells compared with control cells when cells were treated with glutamine deprivation or BPTES (Fig. 3d; Supplementary Fig. 7).

**Fig. 4:**
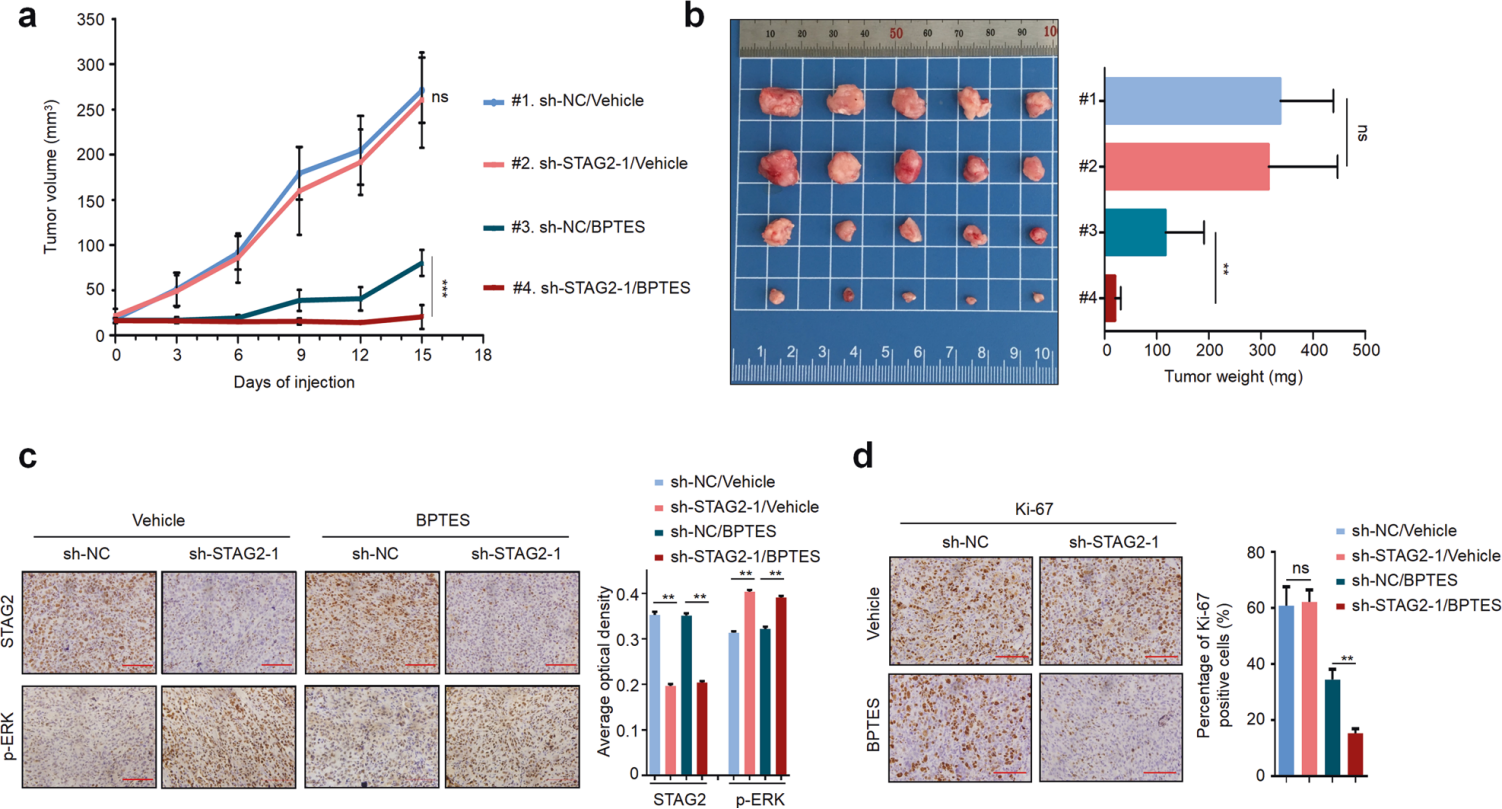
STAG2 knockdown enhances the sensitivity of BRAF-mutant thyroid cancer cells to glutaminase inhibitor in vivo. **a** Growth curves of STAG2-knockdown xenograft tumors and control tumors with the indicated treatments (*n* = 5/group). **b** Left and right panels show the images of dissected tumors and mean tumor weight from the indicated groups, respectively. **c** Representative tumor sections were subjected to IHC staining using STAG2 and phosphorylated ERK (p-ERK) antibodies with quantitative analysis using AOD value. Scale bar, 200 μm. **d** IHC staining of Ki-67 in tumor tissues from the indicated groups. Representative tumor sections are shown in the left panels and the percentage of Ki-67 positive cells are calculated in the right panel from 5 microscopic fields in each group. Data are presented as mean ± SD. ns no significance; **P < 0.01; ***P < 0.001.

We further evaluate the therapeutic potential of BPTES in STAG2-deficient thyroid cancers using xenograft tumor model. The results showed that STAG2 knockdown had no effect on tumor growth in nude mice bearing 8505C-derived tumor when treated with vehicle, while caused a significant reduce in tumor growth compared with the control when treated with BPTES (Fig. 4a, b). Next, we performed IHC assay to confirm the inhibition of STAG2 expression and the enhancement of ERK signaling in STAG2-knockdown tumors (Fig. 4c). Meanwhile, STAG2 knockdown did not cause a significant change in the percentage of Ki-67-positive cells in DMSO-treated tumors, while significantly decreased the percentage of Ki-67-positive cells in BPTES-treated tumors (Fig. 4d). Besides, we evaluated the level of cleaved-PARP, a pro-apoptotic marker, in xenograft tumors by IHC. As shown in Supplementary Fig. 8, we did not find a significant difference between STAG2-knockdown tumors and control tumors when mice were treated with DMSO; however, STAG2-knockdown tumors exhibited a higher level of cleaved-PARP than control tumors when mice were treated with BPTES. These findings indicate that STAG2-deficient thyroid cancer cells are more sensitive to glutamine deprivation or glutaminase inhibitor.

### STAG2 knockdown causes a reduction in the expression of glutamine transporter and glutaminases and an increase in the levels of intracellular ROS

**Fig. 5:**
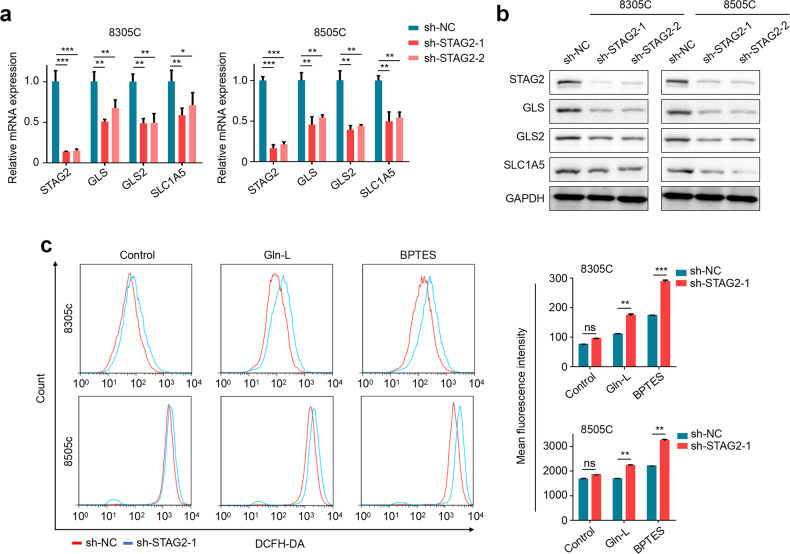
The effects of STAG2 knockdown on the expression of glutamine transporter and glutaminases and cellular ROS levels. The effect of STAG2 knockdown on mRNA and protein expression of GLS, GLS2 and SLC1A5 was analyzed by qRT-PCR (**a**) and western blot (**b**) assays. β-actin was used as an internal reference for the former, and GAPDH was used as a loading control for the latter. **c** STAG2-knockdown 8305C and 8505C cells and their control cells were cultured in the normal medium containing 2 mM glutamine or 50 nM of glutamine (Gln-L) or were treated with 8 μM BPTES for 48 h in the normal medium. Cell were then stained with 20 μM DCFH-DA for 60 min. Next, intracellular ROS levels were quantified by flow cytometry. Left panels show representative flow cytometry profiles and mean fluorescence intensity is quantified in the right panels. Data are presented as mean ± SD. ns none significance; **P < 0.01; **P < 0.01; ***P < 0.001.

To elucidate the mechanism of STAG2-deficient thyroid cancer cells being more sensitive to glutamine deprivation, we first determined the impact of STAG2 knockdown on the expression of glutamine transporter SLC1A5 and glutaminases GLS and GLS2, which are essential for glutamine metabolism [19–21]. The results showed that knocking down STAG2 in 8305C and 8505C markedly decreased mRNA and protein levels of SLC1A5, GLS and GLS2 compared with the control (Fig. 5a, b; Supplementary Fig. 9). Considering that asparagine synthetase (ASNS) is a compensation in glutamine metabolic disorder [22], we thus determined the effect of STAG2 knockdown on ASNS expression, and found increased expression of ASNS in STAG2-knockdown cells (Supplementary Fig. 10), further highlighting the metabolic plasticity of cancer cells.

There are numerous studies indicating that glutamine metabolism is crucial for cellular ROS homeostasis and excessively elevated ROS levels can induce cell death [20, 23, 24]. Thus, we used DCFH-DA probe to detect intracellular ROS. The results showed that, in comparison with the control, STAG2 knockdown had no effect on ROS production when treated with DMSO, while significantly elevated cellular ROS levels when treated with BPTES or glutamine deprivation (Fig. 5c). It is well documented that glutamine metabolism plays a key role in the synthesis of glutathione, which is an important antioxidant in the clearance of ROS [25, 26]. Intracellular total glutathione includes reduced glutathione (GSH) and oxidized glutathione (GSSG). The ratio of the two is often used to indicate the relative antioxidant state of the cell [27]. Thus, we explored the balance of GSH/GSSG in different conditions. The results showed that, compared with the control, knocking down STAG2 in 8305C and 8505C cells almost did not affect the ratio of GSH/GSSG, while significantly decreased this ratio upon glutamine deprivation or BPTES treatment (Supplementary Fig. 11). These results, taken together, indicate that STAG2 inactivation may impair the ability of BRAF-mutant thyroid cancer cells to utilize glutamine and decrease the antioxidant capacity and ROS clearance of cells under glutamine stress condition.

### STAG2 knockdown decreases protein stability of c-Myc by the ERK/AKT/GSK3β feedback axis

**Fig. 6:**
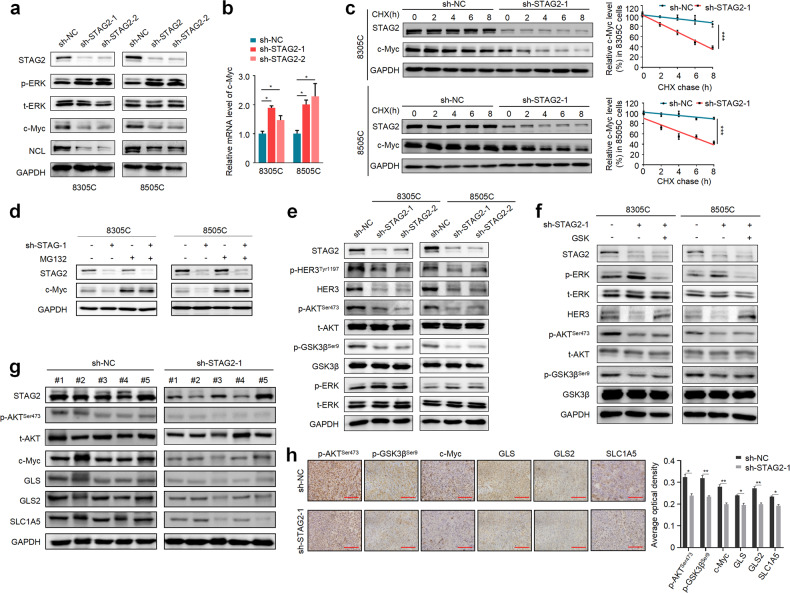
The effect of STAG2 knockdown on the expression and protein stability of c-Myc. **a** Western blot analysis of p-ERK, t-ERK, c-Myc and its downstream target NCL in STAG2-knockdown 8305C and 8505C cells and their control cells. **b** mRNA expression c-Myc was analyzed by qRT-PCR in the above cells. β-actin was used as an internal reference. **c** STAG2-knockdown 8305C and 8505C cells and their control cells were seeded and then treated with 200 μg/mL cycloheximide (CHX) for the indicated times. Next, lysates were prepared and then immunoblotted for the indicated proteins (left panels). The band intensity of c-Myc was normalized to that of GAPDH, and subsequently normalized to that in the DMSO-treated cells (right panels). **d** STAG2-knockdown 8305C and 8505C cells and their control cells were treated with 25 μM MG-132 4 h before harvesting, and then subjected to western blot analysis using the indicated antibodies. **e** The effect of STAG2 knockdown on the expression of HER3 and its downstream molecules was determined by western blot analysis using the indicated antibodies. **f** Western blot analysis of the indicated proteins in STAG2-knockdown 8305C cells and control cells treated with or without MEK inhibitor GSK1120212 (GSK). **g** Lysates from STAG2-knockdown and control tumors were subjected to western blot analysis using the indicated antibodies. **h** Representative tumor sections from STAG2-knockdown and control mice were subjected to IHC assay using the indicated antibodies with quantitative analysis using AOD value. Scale bar, 200 μm. Data are presented as mean ± SD. *P < 0.05; **P < 0.01; ***P < 0.001.

We next aimed to explore the mechanism of which STAG2 regulates the expression of glutamine transporter and glutaminases. The evidences have indicated that c-Myc can regulate glutamine metabolism by inducing the transcription of genes encoding glutamine transporter or glutaminases [28–30]. Thus, we assessed the impact of STAG2 inactivation on c-Myc expression. The results showed that STAG2 knockdown expectedly up-regulated the levels of p-ERK, but down-regulated protein expression of c-Myc and its downstream target nucleolin (NCL) compared with the control (Fig. 6a; Supplementary Fig. 12a). However, we found that STAG2 knockdown increased mRNA expression of c-Myc (Fig. 6b), indicating that STAG2 regulates c-Myc expression at post-transcriptional levels.

Considering that the change of protein stability is one of major determinant factors affecting protein expression [31, 32], we first treated cells with CHX to block de novo protein synthesis, and then assessed its effect on protein stability of c-Myc. The results showed that STAG2 knockdown in these cells significantly accelerated the turnover of c-Myc protein compared with the control (Fig. 6c). Next, we inhibited the ubiquitin-dependent proteasome pathway with MG132, which showed that MG132 partially reversed the inhibitory effect of STAG2 knockdown on protein expression of c-Myc (Fig. 6d; Supplementary Fig. 12b), indicating that STAG2 inactivation promotes protein degradation of c-Myc via the ubiquitin-proteasome pathway.

Increasing evidences have indicated that the overactivation of ERK signaling can inhibit the transcription of *HER3* and the phosphorylation of its downstream effector AKT [12]. Meanwhile, AKT/GSK-3β cascade has also been demonstrated to regulate protein stability of c-Myc [33]. As supported, our data showed that knocking down STAG2 in thyroid cancer cells clearly up-regulated p-ERK levels compared with the control, accompanied by a reduction in HER3 expression and the levels of phosphorylated HER3^Tyr1197^ (p-HER3^Tyr1197^), phosphorylated AKT^Ser473^ (p-AKT^Ser473^) and phosphorylated GSK3β^Ser9^ (p-GSK3β^Ser9^) (Fig. 6e; Supplementary Fig. 12c), and these effects could be partially reversed by MEK inhibitor GSK1120212 (Fig. 6f; Supplementary Fig. 12d). The above conclusions were further supported by in vivo data from the DMSO-treated xenograft tumors showing that the levels of p-AKT^Ser473^ and p-GSK3β^Ser9^ and the expression of c-Myc, SLC1A5, GLS and GLS2 in STAG2-knockdown tumors were obviously decreased compared with control tumors (Fig. 6g, h; Supplementary Fig. 12e). These data, taken together, suggest that STAG2 inactivation reduces protein stability of c-Myc via the ERK/AKT/GSK3β feedback axis, thereby reprograming glutamine metabolism of BRAF-mutant thyroid cancer cells.

### STAG2 inactivation renders BRAF-mutant thyroid cancer cells more sensitive to glutaminase inhibitor by suppressing c-Myc expression

**Fig. 7:**
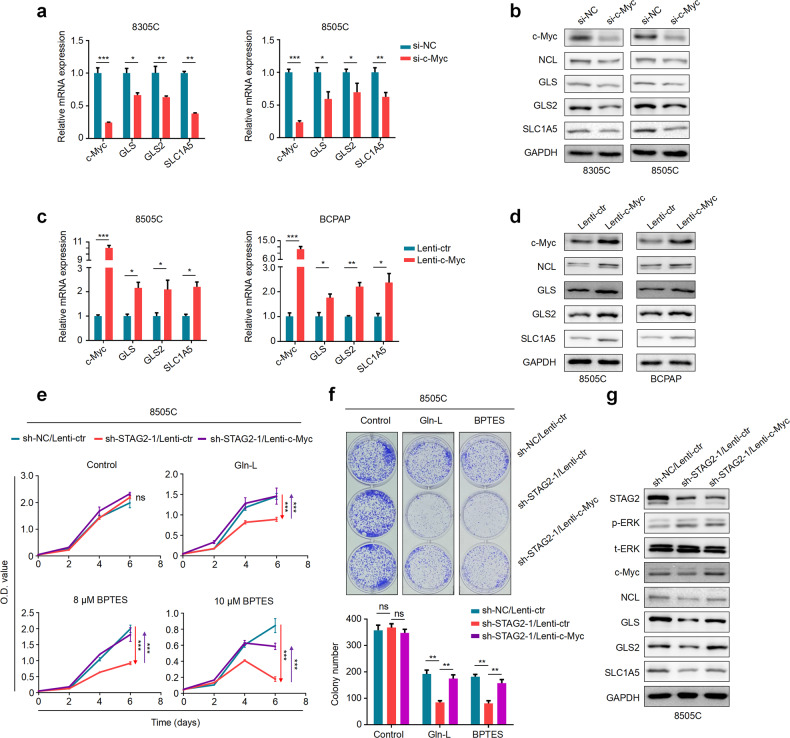
STAG2 affects the glutamine dependence of thyroid cancer cells via c-Myc. **a** The effect of c-Myc knockdown on mRNA expression of *NCL*, *GLS*, *GLS2* and *SLC1A5* in 8305C and 8505C cells was determined by qRT-PCR. β-actin was used as an internal reference. **b** The effect of c-Myc knockdown on protein expression of NCL, GLS, GLS2 and SLC1A5 in 8305C and 8505C cells was determined by western blot analysis. GAPDH was used as a loading control. The effects of ectopic expression of c-Myc on mRNA expression of *GLS*, *GLS2* and *SLC1A5* and protein expression of NCL, GLS, GLS2 and SLC1A5 in 8505C and BCPAP cells was determined by qRT-PCR (**c**) and western blot (**d**) assays. β-actin was used as an internal reference for the former, and GAPDH was used as a loading control for the latter. **e** The MTT assay was performed to assess the proliferation ability of 8505C cells with the indicated treatments. **f** Colony formation ability of 8505C cells with the indicated treatments: Control (2 mM of glutamine), Gln-L (50 nM of glutamine) and BPTES (8 μM BPTES in the medium containing 2 mM of glutamine). The upper panels show representative images and colony number is quantified in the lower panel. **g** Western blot analysis of the indicated proteins in 8505C cells transfected with different lentiviruses. GAPDH was used as a loading control. Data were presented as mean ± SD of values from three different experiments. ns, none significance; *P < 0.05; **P < 0.01; ***P < 0.001.

We first knocked down c-Myc in 8305C and 8505C cells and found that c-Myc knockdown significantly down-regulated mRNA levels of *GLS*, *GLS2* and *SCL1A5* (Fig. 7a) and their protein levels (Fig. 7b; Supplementary Fig. 13a) in comparison with the control. Conversely, we ectopically expressed c-Myc in 8505C and BCPAP cells and confirmed its regulator effect on the expression of NCL, GLS, GLS2 and SCL1A5 by qRT-PCR and western blot assays (Fig. 7c, d; Supplementary Fig. 13b).

c-Myc has been proved to regulate the expression of SLC1A5 and GLS at transcriptional or post-transcriptional levels [28, 34], however, it is not clear whether GLS2 can serve as a potential downstream target of c-Myc. Thus, we first predicted potential binding sites of c-Myc in the promoter region of *GLS2* using online tool (http://jaspar.genereg.net/). Next, dual-luciferase reporter assay was performed in 293 T cells transfected with pGL3-GLS2-Luc plasmid. The results showed that ectopic expression of c-Myc significantly increased promoter activity of *GLS2* compared with the control (Supplementary Fig. 14a). Further ChIP-qPCR assay was performed to target its different promoter regions in 293 T cells using anti-Myc antibody. The results showed that three fragments (P1: -752/-564; P2: -578/-347; P4: +334/+531) within *GLS2* promoter were enriched in c-Myc overexpressing 293 T cells compared to control cells (Supplementary Fig. 14b). Similar results were also found in 8505C and BCPAP cells (Supplementary Fig. 15). These observations indicate that GLS, GLS2 and SLC1A5 may be downstream targets of c-Myc.

To determine whether STAG2 inactivation enhancing the response of thyroid cancer cells to glutamine deprivation was mediated by c-Myc, we ectopically expressed c-Myc in STAG2-knockdown thyroid cancer cells. As shown in Fig. 7e and Supplementary Fig. 16a, STAG2 knockdown significantly suppressed cell proliferation when cells were subjected to glutamine inhibition, and this effect could be effectively reversed by ectopic expression of c-Myc. Colony formation and apoptotic assays further supported the above conclusion (Fig. 7f; Supplementary Fig. 16b). Meanwhile, ectopic expression of c-Myc in STAG2-knockdown 8505C cells attenuated the pro-apoptosis effect of glutamine deprivation and BPTES treatment (Supplementary Fig. 17). Besides, we also found that ectopic expression of c-Myc in STAG2-knockdown cells reversed the inhibitory effect of STAG2 knockdown on the expression of GLS, GLS2 and SLC1A5 (Fig. 7g; Supplementary Fig. 18).

**Fig. 8:**
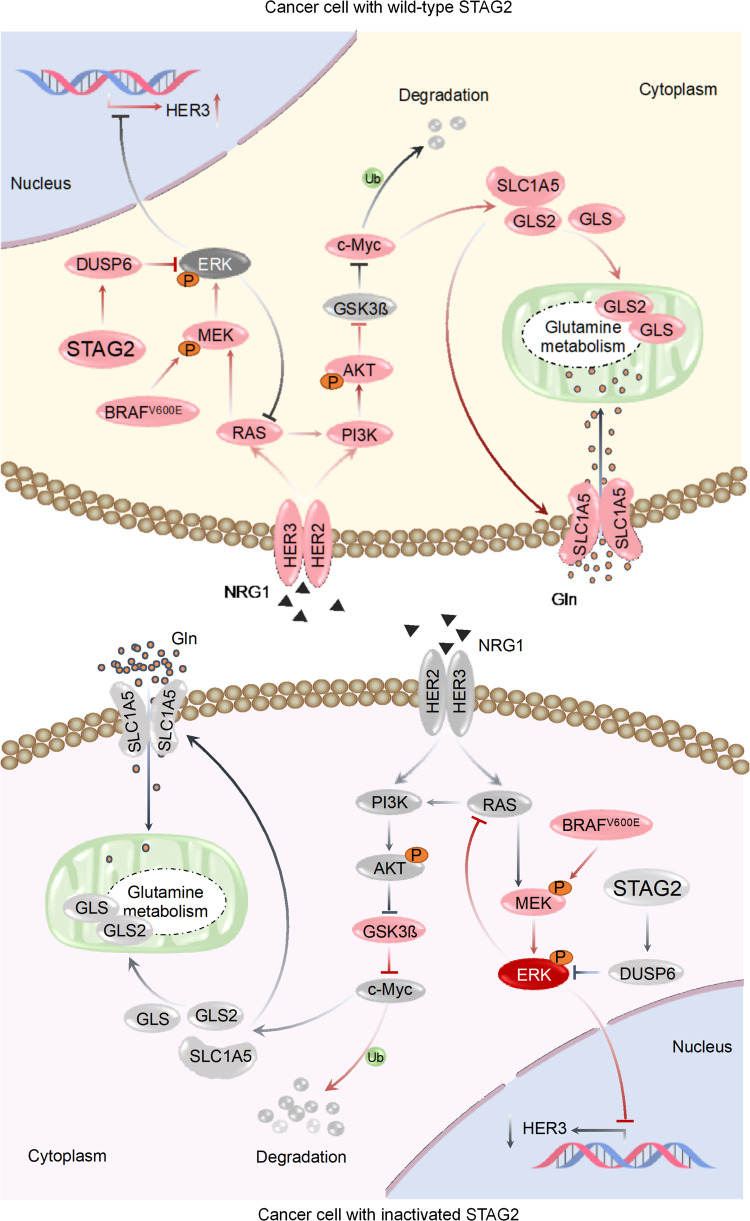
A schematic model of which STAG2 inactivation reprograms glutamine metabolism of BRAF-mutant thyroid cancer cells. Usually, STAG2 inhibits ERK signaling by increasing DUSP6, while this signaling is overactivated upon STAG2 inactivation in BRAF-mutant thyroid cancer cells. Activated ERK promotes AKT/GSK3β signaling-mediated protein degradation of c-Myc via feedback suppression of HER3 transcription and RAS signaling. As a result, c-Myc downstream glutamine transporter and glutaminases such as GLS, GLS2 and SLC1A5 are down-regulated, thereby leading to reprogramming of glutamine metabolism and making BRAF-mutant thyroid cancer cells more sensitive to glutamine deprivation and glutaminase inhibitor.

From the above results, we proposed a model to elucidate the mechanism of which STAG2 inactivation reprograms glutamine metabolism of BRAF-mutant thyroid cancer cells (Fig. 8). Specifically, ERK cascade is overactivated when STAG2 is down-regulated in thyroid cancer cells, followed with suppression of *HER3* transcription and RAS signaling to promote protein degradation of c-Myc via AKT/GSK3β signaling pathway. As a result, glutamine metabolism of BRAF-mutant thyroid cancer cells is reprogrammed due to down-regulation of c-Myc downstream glutamine transporter and glutaminases, thereby making these cells more sensitive to glutamine deprivation and glutaminase inhibitor. Thus, targeting glutamine metabolism will provide an alternative therapeutic strategy for STAG2-deficient thyroid cancers.

## Discussion

STAG2 is one of the most important subunits of cohesin complex [13, 35], and the most commonly mutated subunit [36]. Inactivating mutations of *STAG2* cause cohesin defects and aneuploidy in some kinds of cancer cells [14], but not all tumor-derived *STAG2* mutations confer defects in aneuploidy, indicating that loss of STAG2 may contribute to the pathogenesis or progression or therapy of human cancers by other unknown mechanisms. As supported, a previous study demonstrated that, although STAG2 inactivation did not affect the proliferation of BRAF-mutant melanoma cells, it markedly attenuated their response to RAF/MEK inhibitors by suppressing CCCTC-binding-factor (CTCF)-mediated expression of DUSP6 and subsequently reactivating ERK signaling [15].

In the present study, we found that, although there was only one mutation in *STAG2* gene, its expression was significantly down-regulated in PTCs. Unlike BRAF-mutant melanoma cells, STAG2 knockdown in BRAF-mutant thyroid cancer cells did not affect their cellular response to MEK inhibitor. This difference may be attributed to feedback activation of *HER3* transcription by RAF or MEK inhibitors in BRAF-mutant thyroid cancers [12], but not in BRAF-mutant melanomas. In addition, many other resistance mechanisms have also been identified in ERK-activated tumors such as melanoma, thyroid cancer and colorectal cancer, mostly through the reactivation of MAPK/ERK pathway or bypass activation of PI3K/AKT pathway [37, 38]. However, the difference between BRAF-mutated melanomas and thyroid cancers on STAG2 inactivation-induced resistance mechanism still need to be further investigated.

Energy metabolism as a fundamental process is critical for cellular health and function. Unlike normal cells, cancer cells usually reprogram their metabolism to fulfill energy requirements [17]. Considering that glucose and glutamine are major energy sources for cancer cell growth [39], energy stress experiment was carried out in our study. Our data showed that STAG2 knockdown not only made BRAF-mutant thyroid cancer cells more dependent on glutamine rather than glucose, but also rendered them more sensitive to glutaminase inhibitor. As supported, we found that STAG2 knockdown in these cells significantly reduced the expression of glutamine transporter SLC1A5 and glutaminases GLS and GLS2 at both mRNA and protein levels. The former is essential for glutamine uptake [40], while the latter two can catalyze the hydrolysis of glutamine to glutamate and ammonia [41, 42]. In addition, our data also indicated that STAG2 knockdown in BRAF-mutant thyroid cancer cells led to an elevated ROS level and a declined GSH/GSSH ratio when glutamine deprivation or treated with glutaminase inhibitor, meaning that the cells are less resistant to oxidative stress damage due to their reduced ability to use glutamine [25, 26].

Previous studies have confirmed that both SLC1A5 and GLS can be regulated by c-Myc in different ways [28, 34], also supported by the present study. Besides, we identified GLS2 as a direct downstream target of c-Myc, which was in line with a previous study showing that GLS2 was regulated by c-Myc in activated CD4 + T cells [43]. Thus, we speculate that c-Myc may act as a key player in STAG2 inactivation-mediated reprogramming of glutamine metabolism in BRAF-mutant thyroid cancer cells. As supported, our data showed that, STAG2 post-transcriptionally regulated c-Myc expression, and ectopic expression of c-Myc reversed the inhibitory action of STAG2 knockdown on the expression of GLS, GLS2 and SLC1A5, meanwhile, attenuated the inhibitory effect of STAG2 inactivation on cell proliferation by glutamine deprivation.

c-Myc protein stability is regulated by two adjacent N-terminal phosphorylation sites, Thr-58 and Ser-62, that exhibit opposing roles in the control of its stability [32]. RAS/RAF//MEK/ERK signaling pathway increases protein stability of c-Myc via phosphorylation at Ser-62, while RAS/PI3K/AKT signaling pathway stabilizes c-Myc by phosphorylating and inactivating GSK3β and subsequently reducing c-Myc phosphorylation at Thr-58. Notably, phosphorylation of Thr-58 is generally dependent on a prior phosphorylation of Ser-62 [32, 44]. Our data demonstrated that STAG2 inactivation led to overactivation of ERK signaling and subsequent feedback inhibition of AKT signaling, thereby reducing protein stability of c-Myc and reprograming glutamine metabolism, further supporting the above conclusions.

Glutamine addiction can be seen in some kinds of tumors that are c-Myc dependent or overactivation of glutaminolysis [34, 45]. In these cases, glutaminolysis occupies the core of energy metabolism, which acts as important as or even more important than glycolysis. On the other hand, glutamine belongs to a group of amino acids that are conditionally essential because it is required for biological synthesis and energy support especially in cancer cells, although it can be readily synthesized endogenously [46]. There is a basis requirement for glutamine supplement, which also depend on the capacity of utilizing glutamine. STAG2-deficient thyroid cancer cells exhibit more lower glutamine availability due to decreased levels of c-Myc. Notably, the present study found that ASNS was elevated in STAG2-knockdown cells compared with control cells, indicating that there may be a metabolic compensatory effect in STAG2-deficient cells. There is evidence demonstrating that ASNS synthesizes de novo asparagine from aspartate and glutamine, being required to suppress inadequate metabolism of glutamine-induced apoptosis [47, 48]. Even so, when exogenetic glutamine is deprived or glutaminases are inhibited, the above compensatory mechanism is also insufficient to maintain the proliferation and survival of STAG2-knockdown cells, eventually leading to their death. However, whether targeting ANSN may be an alternative therapeutic strategy for STAG2-deficient thyroid cancer cells need be further explored.

In summary, the present study demonstrates that, although STAG2 inactivation activates ERK signaling, it does not affect cell proliferation and colony formation of BRAF-mutant thyroid cancer cells as well as their response to MEK inhibitor. Surprisingly, we find that STAG2 knockdown makes BRAF-mutant thyroid cancer cells more sensitive to glutamine deprivation or glutaminase inhibitor via the ERK/AKT/GSK3β/c-Myc feedback axis. Thus, targeting glutamine metabolism will provide an alternative therapeutic strategy for STAG2-deficient thyroid cancers.

## Materials and methods

### Clinical samples and Immunohistochemistry (IHC)

A total of 13 pairs of paraffin embedding surgical PTCs and matched non-cancerous tissues were obtained from the First Affiliated Hospital of Xi’an Jiaotong University. All of the tissues were histologically examined by two senior pathologists at Department of Pathology of the Hospital based on World Health Organization (WHO) criteria. The study was conducted in accordance with the Declaration of Helsinki, and the protocol was approved by the Institutional Review Board and Human Ethics Committee of the First Affiliated Hospital of Xi’an Jiaotong University. The IHC staining for clinical samples was carried out as described previously [49]. Protein expression was quantitated by Average Density (Average Optical Density, AOD, calculated as intense optical density/area) using ImageJ software (version 1.53).

### Cell lines and drug treatments

*BRAF*^*V600E*^-mutant human ATC cell lines 8305C and 8505C as well as PTC cell line BCPAP were kindly provided by Dr. Haixia Guan (Guangdong Provincial People’s Hospital, Guangzhou, P.R. China). *BRAF*^*V600E*^-mutant human melanoma cell line A375 was obtained from ATCC (Rockville, MD). Of them, 8305C, 8505C and BCPAP cells were cultured in RPMI 1640 medium with 10% fetal bovine serum (FBS), while A375 cells were cultured in DMEM medium with 10% FBS. We exclude the mycoplasma contamination using the One-step Quickcolor Mycoplasma Detection Kit (Shanghai Yise Medical Technology Co., Ltd). In some experiments, the medium was prepared by adding extremely low concentration (10 nM, 50 nM or 200 nM) of glutamine into glutamine-free RPMI-1640 medium supplemented with 10% FBS, compared with normal glutamine concentration medium (2 mM). Cells were treated with selective BRAF kinase inhibitor PLX4032, MEK inhibitor GSK1120212, c-Myc inhibitor 10074-G5, glutaminase inhibitor Bis-2-(5-phenylacetamido-1,3,4-thiadiazol-2-yl)ethyl sulfide (BPTES), proteasome inhibitor MG132 or cycloheximide (CHX) at the indicated concentrations and times, and the medium and agent were replenished every 24 h. PLX4032, GSK1120212, BPTES and MG132 were purchased from Selleck Chemicals (Houston, TX), and CHX was purchased from MP Biomedicals (Santa Ana, CA), which were all dissolved in dimethyl sulfoxide (DMSO), sub-packaged and stored at -80°C until use. Equal volume of vehicle was used as the control.

### Lentivirus transfection and short interfering RNAs (siRNAs)

Lentivirus negative control (sh-NC) and STAG2-shRNAs (sh-STAG2-1 and -2) were obtained from HanBio Biotechnology Co., Ltd (Shanghai, P.R. China). Lentivirus encoding c-Myc and control lentivirus (GV358) were purchased form Genechem chemical technology co., LTD (Shanghai, P.R. China). shRNAs targeting sequences were presented in Table S1. Cells were transfected at 30–40% confluence with a final lentivirus multiplicity of infection (MOI) of 50 for shRNAs. After 48 h transfection, puromycin was added to pick out stable-transfected cells.

Oligonucleotides of siRNA targeting c-Myc and control siRNA (Table S1) were obtained from RiboBio Co., Ltd (Guangzhou, P.R. China). Cells were plated on a six-well plate to achieve 50% confluence and then transfected with the above siRNAs at a final concentration of 60 nM using X-tremeGENE siRNA Transfection Reagent (Roche Diagnostics, Mannheim, Germany).

### Cell proliferation and drug inhibition test

Cells with appropriate density were seeded in 96-well plates and cultured with corresponding medium, various concentrations of glutamine medium or relevant drugs for appropriate time. The MTT assay was then performed to evaluate cell proliferation as previously described [50]. IC_50_ (half maximal inhibitory concentration) values were calculated as described previously [49]. Three triplicates were done to determine each data point.

### Colony formation assay

Cells were seeded in 12-well plates at a density of 2000–3000 cells/mL and treated with corresponding medium and indicated concentration of drugs for 7–10 days. The medium and agent were replenished every 48 h. Cells were then fixed with methanol and stained with crystal violet. Three parallel experiments were done.

### Cell apoptosis assay

Cells were treated with normal medium, non-glutamine medium or 8 μM BPTES for 48 h, then harvested, washed with phosphate-buffered saline (PBS) and stained with Annexin V-FITC Detection Kit (Roche Applied Science, Penzberg, Germany) according to the manufacturer’s protocol. The apoptotic cells were analyzed by a Flow Cytometer (BD Biosciences, Franklin Lakes, NJ). Each experiment was performed in triplicate.

### Measurement of cellular ROS levels

Cellular ROS levels were detected using 2’, 7’-dichlorodihydro-fluorescein diacetate (DCFH-DA; Sigma). Cells were seeded in 6-well plates and treated with non-glutamine medium, 8 μM BPTES or DMSO for 48 h. Cells were then stained with 10 μM DCFH-DA for 1 h, then harvested, washed and tested by flow cytometry. Each experiment was done in triplicate.

### Measurement of reduced GSH/oxidized glutathione (GSSG) ratio

Cells were cultured in the medium containing 2 mM or 50 nM of glutamine or were treated with 8 μM BPTES for 24 h. Intracellular total GSH and GSSG were then measured using a GSH and GSSG Detection Assay Kit (S0053, Beyotime Biotechnology, China) according to the manufacturer’s protocol. Next, the GSH/GSSG ratio was calculated as ([total GSH] − 2×[GSSG])/[GSSG]. Each experiment was run in triplicate.

### RNA extraction and qRT-PCR

Using TRIZOL reagent (Takara, Inc., Dalian, P.R. China), total RNA was isolated from cell lines and then converted to cDNA with PrimeScript RT reagent Kit (Takara, Inc., Dalian, P.R. China) according to the manufacturer’s protocol. qRT-PCR was carried out on a CFX96 Thermal Cycler Dice^TM^ real-time PCR system (Bio-Rad Laboratories, Hercules, CA) using SYBR Premix Ex Taq^TM^ (Takara, Inc., Dalian, P.R. China). The primer sequences were presented in Table S2. β-actin was used as an internal reference. Three triplicates were done for each sample.

### Western blot analysis

Cells were washed with PBS and lysed in prechilled RIPA buffer containing protease inhibitors. Xenograft tumor tissues were homogenized in TissueLyser LT (Qiagen, Hilden, Germany) with RIPA buffer containing protease inhibitors. Equal amounts of protein lysates were separated by SDS-PAGE and transferred to PVDF membranes (Roche Diagnostics, Mannheim, Germany). The membranes were then incubated overnight with the following primary antibodies listed in Table S3. This was followed by incubation with their respective HRP-conjugated secondary antibodies from ZSGB-BIO and immunoblotting signals were visualized using the Western Bright ECL detection system (Advansta, Inc., Menlo Park, CA). The quantitative analysis of band intensity normalized to loading control was performed by ImageJ software.

### Cycloheximide (CHX) chase assay

Cells were treated with 200 μg/mL CHX to stop de novo protein synthesis in STAG2-knockdown cells or control cells. At the indicated time points, cell lysates were harvested and then subjected to immunoblotting.

### Dual-luciferase reporter system

The promoter region of *GLS2* gene was inserted into pre-digested pGL3-Basic luciferase vector (Promega) to produce the luciferase reporter plasmids pGL3-GLS2-Luc. The constructs were verified by Sanger sequencing. The primers for plasmid constructs were presented in Table S4. Next, c-Myc-overexpression 293 T cells and control cells were co-transfected with pGL3-GLS2-Luc and pRL-TK plasmids (Promega) using X-tremeGENE HP DNA Transfection Reagent (Roche). Cells were collected 48 h post-transfection, and luciferase activities were then analyzed by the dual-luciferase reporter system (Promega). Data were expressed as relative luciferase activity (Firefly luciferase activity/Renilla luciferase activity).

### Chromatin immunoprecipitation (ChIP)-quantitative PCR (qPCR)

The ChIP assay was used to determine the binding of c-Myc to its target DNA as described previously [51]. The DNA fragments were then used as templates for qPCR analysis using the primers presented in Table S5. Data were normalized by respective 10% input. Each experiment was done in triplicate.

### Animal studies

Four- to five-week-old female BALB/c athymic mice were purchased from SLAC Laboratory Animal Co., Ltd. (Shanghai, P.R. China) and were then injected subcutaneously with STAG2-knockdown 8505C cells (1 × 10^7^) or control cells into their groin regions. When tumors grew to around 5 mm in diameter, mice were randomly divided into four groups (five mice per group). BPTES (10 mg/kg) or vehicle was then administered by intraperitoneal injection every other three days. During this time, tumor size was measured every two days and calculated tumor volume. All mice were sacrificed 2 h after the last treatment. Tumors were then harvested and weighted. Part of xenograft tumors were embedded in paraffin for subsequent IHC assay as previously described [51]. Protein expression was quantitated by AOD using ImageJ software (version 1.53). The remaining tumors were used for western blot analysis. All animal experiments were conducted in accordance with Institution Guidelines and approved by the Laboratory Animal Center of Xi’an Jiaotong University.

### Statistical analysis

Independent *t* test, paired *t* test and two-way ANOVA were used for comparing the data (SPSS Statistics for Windows v16.0, Chicago, IL). To achieve statistical significance for differences, all in vitro experiments were conducted in triplicate and repeated three times. A *P*-value of <0.05 was considered to be statistically significant. Unless indicated, the data shown in the figures are representative examples.

## Supplementary information

Supplementary information

Original Data File

aj-checklist

## Data Availability

All data generated or analyzed during the current study are available from the corresponding author on reasonable request.

## References

[1] Rahib L, Smith BD, Aizenberg R, Rosenzweig AB, Fleshman JM, Matrisian LM (2014). Projecting cancer incidence and deaths to 2030: the unexpected burden of thyroid, liver, and pancreas cancers in the United States. Cancer Res.

[2] Zheng R, Zhang S, Zeng H, Wang S, Sun K, Chen R (2022). Cancer incidence and mortality in China, 2016. J Natl Cancer Cent..

[3] Siegel RL, Miller KD, Jemal A (2020). Cancer statistics, 2020. CA Cancer J Clin.

[4] Cabanillas ME, Mcfadden DG, Durante C (2016). Thyroid cancer. Lancet..

[5] Schmidbauer B, Menhart K, Hellwig D, Grosse J (2017). Differentiated thyroid cancer-treatment: state of the art. Int J Mol Sci.

[6] Landa I, Ibrahimpasic T, Boucai L, Sinha R, Knauf JA, Shah RH (2016). Genomic and transcriptomic hallmarks of poorly differentiated and anaplastic thyroid cancers. J Clin Invest.

[7] Liu D, Hu S, Hou P, Jiang D, Condouris S, Xing M (2007). Suppression of BRAF/MEK/MAP kinase pathway restores expression of iodide-metabolizing genes in thyroid cells expressing the V600E BRAF mutant. Clin Cancer Res..

[8] Durante C, Puxeddu E, Ferretti E, Morisi R, Moretti S, Bruno R (2007). BRAF mutations in papillary thyroid carcinomas inhibit genes involved in iodine metabolism. J Clin Endocrinol Metab..

[9] Tao Y, Wang F, Shen X, Zhu G, Liu R, Viola D (2021). BRAF V600E Status sharply differentiates lymph node metastasis-associated mortality risk in papillary thyroid cancer. J Clin Endocrinol Metab.

[10] Xing M (2007). BRAF mutation in papillary thyroid cancer: pathogenic role, molecular bases, and clinical implications. Endocr Rev.

[11] Chavda J, Bhatt H (2020). Systemic review on B-Raf(V600E) mutation as potential therapeutic target for the treatment of cancer. Eur J Med Chem.

[12] Montero-Conde C, Ruiz-Llorente S, Dominguez JM, Knauf JA, Viale A, Sherman EJ (2013). Relief of feedback inhibition of HER3 transcription by RAF and MEK inhibitors attenuates their antitumor effects in BRAF-mutant thyroid carcinomas. Cancer Discov.

[13] Losada A (2014). Cohesin in cancer: chromosome segregation and beyond. Nat Rev Cancer.

[14] Solomon DA, Kim T, Diaz-Martinez LA, Fair J, Elkahloun AG, Harris BT (2011). Mutational inactivation of STAG2 causes aneuploidy in human cancer. Science.

[15] Shen CH, Kim SH, Trousil S, Frederick DT, Piris A, Yuan P (2016). Loss of cohesin complex components STAG2 or STAG3 confers resistance to BRAF inhibition in melanoma. Nat Med.

[16] Lu W, Pelicano H, Huang P (2010). Cancer metabolism: is glutamine sweeter than glucose?. Cancer Cell.

[17] Martinez-Outschoorn UE, Peiris-Pages M, Pestell RG, Sotgia F, Lisanti MP (2017). Cancer metabolism: a therapeutic perspective. Nat Rev Clin Oncol.

[18] Chen L, Cui H (2015). Targeting glutamine induces apoptosis: a cancer therapy approach. Int J Mol Sci.

[19] Deberardinis RJ, Mancuso A, Daikhin E, Nissim I, Yudkoff M, Wehrli S (2007). Beyond aerobic glycolysis: transformed cells can engage in glutamine metabolism that exceeds the requirement for protein and nucleotide synthesis. Proc Natl Acad Sci USA.

[20] Hensley CT, Wasti AT, Deberardinis RJ (2013). Glutamine and cancer: cell biology, physiology, and clinical opportunities. J Clin Invest.

[21] Reckzeh ES, Karageorgis G, Schwalfenberg M, Ceballos J, Nowacki J, Stroet M (2019). Inhibition of glucose transporters and glutaminase synergistically impairs tumor cell growth. Cell Chem Biol.

[22] Bodineau C, Tome M, Murdoch P, Duran RV (2022). Glutamine, MTOR and autophagy: a multiconnection relationship. Autophagy..

[23] Kong B, Qia C, Erkan M, Kleeff J, Michalski CW (2013). Overview on how oncogenic Kras promotes pancreatic carcinogenesis by inducing low intracellular ROS levels. Front Physiol.

[24] Mukha A, Kahya U, Linge A, Chen O, Lock S, Lukiyanchuk V (2021). GLS-driven glutamine catabolism contributes to prostate cancer radiosensitivity by regulating the redox state, stemness and ATG5-mediated autophagy. Theranostics..

[25] Mates JM, Campos-Sandoval JA, de Los SJ, Marquez J (2020). Glutaminases regulate glutathione and oxidative stress in cancer. Arch Toxicol.

[26] Hayes JD, Dinkova-Kostova AT, Tew KD (2020). Oxidative stress in cancer. Cancer Cell.

[27] Griffith OW (1999). Biologic and pharmacologic regulation of mammalian glutathione synthesis. Free Radic Biol Med.

[28] Gao P, Tchernyshyov I, Chang TC, Lee YS, Kita K, Ochi T (2009). c-Myc suppression of miR-23a/b enhances mitochondrial glutaminase expression and glutamine metabolism. Nature..

[29] Liu W, Le A, Hancock C, Lane AN, Dang CV, Fan TW (2012). Reprogramming of proline and glutamine metabolism contributes to the proliferative and metabolic responses regulated by oncogenic transcription factor c-MYC. Proc Natl Acad Sci USA.

[30] Shroff EH, Eberlin LS, Dang VM, Gouw AM, Gabay M, Adam SJ (2015). MYC oncogene overexpression drives renal cell carcinoma in a mouse model through glutamine metabolism. Proc Natl Acad Sci USA.

[31] Luscher B, Eisenman RN (1986). Proteins encoded by the c-myc oncogene: analysis of c-myc protein degradation. Princess Takamatsu Symp..

[32] Sears RC (2004). The life cycle of C-myc: from synthesis to degradation. Cell Cycle.

[33] Rottmann S, Wang Y, Nasoff M, Deveraux QL, Quon KC (2005). A TRAIL receptor-dependent synthetic lethal relationship between MYC activation and GSK3beta/FBW7 loss of function. Proc Natl Acad Sci USA.

[34] Wise DR, Deberardinis RJ, Mancuso A, Sayed N, Zhang XY, Pfeiffer HK (2008). Myc regulates a transcriptional program that stimulates mitochondrial glutaminolysis and leads to glutamine addiction. Proc Natl Acad Sci USA.

[35] Michaelis C, Ciosk R, Nasmyth K (1997). Cohesins: chromosomal proteins that prevent premature separation of sister chromatids. Cell..

[36] Kim JS, He X, Orr B, Wutz G, Hill V, Peters JM (2016). Intact cohesion, anaphase, and chromosome segregation in human cells harboring tumor-derived mutations in STAG2. PLoS Genet.

[37] Lito P, Rosen N, Solit DB (2013). Tumor adaptation and resistance to RAF inhibitors. Nat Med.

[38] Shi H, Hugo W, Kong X, Hong A, Koya RC, Moriceau G (2014). Acquired resistance and clonal evolution in melanoma during BRAF inhibitor therapy. Cancer Discov.

[39] Deberardinis RJ, Lum JJ, Hatzivassiliou G, Thompson CB (2008). The biology of cancer: metabolic reprogramming fuels cell growth and proliferation. Cell Metab.

[40] Yoo HC, Park SJ, Nam M, Kang J, Kim K, Yeo JH (2020). A variant of SLC1A5 Is a mitochondrial glutamine transporter for metabolic reprogramming in cancer cells. Cell Metab.

[41] Masisi BK, El AR, Alfarsi L, Rakha EA, Green AR, Craze ML (2020). The role of glutaminase in cancer. Histopathology..

[42] Saha SK, Islam S, Abdullah-Al-Wadud M, Islam S, Ali F, Park KS (2019). Multiomics analysis reveals that GLS and GLS2 differentially modulate the clinical outcomes of cancer. J Clin Med.

[43] Wang R, Dillon CP, Shi LZ, Milasta S, Carter R, Finkelstein D (2011). The transcription factor Myc controls metabolic reprogramming upon T lymphocyte activation. Immunity..

[44] Sears R, Nuckolls F, Haura E, Taya Y, Tamai K, Nevins JR (2000). Multiple Ras-dependent phosphorylation pathways regulate Myc protein stability. Genes Dev.

[45] Yuneva M, Zamboni N, Oefner P, Sachidanandam R, Lazebnik Y (2007). Deficiency in glutamine but not glucose induces MYC-dependent apoptosis in human cells. J Cell Biol.

[46] Mates JM, Segura JA, Campos-Sandoval JA, Lobo C, Alonso L, Alonso FJ (2009). Glutamine homeostasis and mitochondrial dynamics. Int J Biochem Cell Biol.

[47] Pavlova NN, Hui S, Ghergurovich JM, Fan J, Intlekofer AM, White RM (2018). As extracellular glutamine levels decline, asparagine becomes an essential amino acid. Cell Metab.

[48] Toda K, Kawada K, Iwamoto M, Inamoto S, Sasazuki T, Shirasawa S (2016). Metabolic alterations caused by KRAS mutations in colorectal cancer contribute to cell adaptation to glutamine depletion by upregulation of asparagine synthetase. Neoplasia..

[49] Wang L, Tian Z, Yang Q, Li H, Guan H, Shi B (2015). Sulforaphane inhibits thyroid cancer cell growth and invasiveness through the reactive oxygen species-dependent pathway. Oncotarget..

[50] Cui B, Yang Q, Guan H, Shi B, Hou P, Ji M (2014). PRIMA-1, a mutant p53 reactivator, restores the sensitivity of TP53 mutant-type thyroid cancer cells to the histone methylation inhibitor 3-Deazaneplanocin A. J Clin Endocrinol Metab.

[51] Qu Y, Yang Q, Liu J, Shi B, Ji M, Li G (2017). c-Myc is Required for BRAF(V600E)-Induced Epigenetic Silencing by H3K27me3 in Tumorigenesis. Theranostics..

