# Supplementary material for: STAG2 inactivation reprograms glutamine metabolism of BRAF-mutant thyroid cancer cells
Source: Cell Death Dis. 2023 Jul 21;14(7):454. doi: 10.1038/s41419-023-05981-z (PMC10361981; doi:10.1038/s41419-023-05981-z)
Supplement: Supplementary file 1 — Supplementary information [file 41419_2023_5981_MOESM1_ESM.docx]

**Supplementary information for**

**STAG2 inactivation reprograms glutamine metabolism of BRAF-mutant thyroid cancer cells**

Xinru Li^1,2^, Yan Liu^1^, Juan Liu^1^, Wei Qiang^3^, Jingjing Ma^1^, Jingyi Xie^1^, Pu Chen^1^, Yubo Wang^1^, Peng Hou^1,*^, and Meiju Ji^4,*^

**Running title:** The regulation of glutamine metabolism by STAG2 in thyroid cancer

***To whom correspondence should be addressed:**

Meiju Ji, Ph.D

Center for Translational Medicine, The First Affiliated Hospital of Xi’an Jiaotong University, Xi’an 710061, China

E-mail: mjji0409@xjtu.edu.cn

**Correspondence may also be addressed**

Peng Hou, Ph.D

Key Laboratory for Tumor Precision Medicine of Shaanxi Province and Department of Endocrinology, The First Affiliated Hospital of Xi’an Jiaotong University, Xi’an 710061, China

E-mail: [phou@xjtu.edu.cn](mailto:phou@xjtu.edu.cn)

**Supplementary tables**

**Supplementary Table 1** shRNAs/siRNAs target sequences in this study

| **Name of the sequence** | **Target sequence (5’-3’)** |
| --- | --- |
| sh-NC | TTCTCCGAACGTGTCACGTAA |
| sh-STAG2-1 | CACTAAATCTTAGCATTAA |
| sh-STAG2-2 | AGAACTTCTTCACTACTCT |
| si-NC | TTCTCCGAACGTGTCACGT |
| si-c-Myc | AACGATTCCTTCTAACAGA |

**Supplementary Table 2** Sequences of qRT-PCR primers used in this study

| **Genes** | **Forward primer (5’-3’)** | **Reverse primer (5’-3’)** |
| --- | --- | --- |
| *STAG2* | TTCTGGAACACCTCAATGTCA | ATCTCTCCATCCCTTCCGAG |
| *c-Myc* | CACCGAGTCGTAGTCGAGGT | TTTCGGGTAGTGGAAAACCA |
| *GLS* | AGGGTCTGTTACCTAGCTTGG | ACGTTCGCAATCCTGTAGATTT |
| *GLS2* | TGCCTATAGTGGCGATGTCTCA | GTTCCATATCCATGGCTGACAA |
| *SLC1A5* | GAGCTGCTTATCCGCTTCTTC | GGGGCGTACCACATGATCC |
| *β-actin* | CCTTGCACATGCCGGAG | GCACAGAGCCTCGCCTT |

**Supplementary Table 3** Antibodies used in this study

| **Antibodies** | **Catalog#** | **Source** |
| --- | --- | --- |
| anti-STAG2 | sc-81852 | Santa Cruz Biotechnology |
| anti-c-Myc | sc-764 | Santa Cruz Biotechnology |
| anti-HER3/ErbB-3 | sc-416 | Santa Cruz Biotechnology |
| anti-p-HER3^Tyr1197^ | ab133444 | Abcam |
| anti-c-Myc (ChIP) | #13987 | Cell Signaling Technology |
| anti-p-ERK | #4370 | Cell Signaling Technology |
| anti-t-ERK | #9102 | Cell Signaling Technology |
| anti-GSK3β | #12456 | Cell Signaling Technology |
| anti-pGSK3β^Ser9^ | #9323 | Cell Signaling Technology |
| anti-pAKT^Ser473^ | BS4007 | Bioworld Technology |
| anti-tAKT | BS1379 | Bioworld Technology |
| anti-GAPDH | AP0063 | Bioworld Technology |
| anti-GLS | D161715 | BBI Life Sciences Corp. |
| anti-SLC1A5 | D260740 | BBI Life Sciences Corp. |
| anti-DUSP6 | ab76310 | Abcam |
| anti-GLS2 | abs110665 | Absin |
| anti-Ki67 | 550609 | BD Pharmingen |
| anti-ASNS | sc-365809 | Santa Cruz Biotechnology |
| anti-c-PARP | #AF7023 | affinity |

**Supplementary Table 4** Sequences of luciferase plasmid construction used in this study

| **Genes** | **Position** | **Forward primer (5’-3’)** | **Reverse primer (5’-3’)** | **Restriction sites** |
| --- | --- | --- | --- | --- |
| *pGL3-GLS2-Luc* | 856/+732 | TACCGAGCTCTTACGCGTGCTAGCTAGAGGGACACAAACTGG | ACAGTACCGGAATGCCAAGCTTCTCTGGTTTGGGAAATCTG | *NheI* & *HindⅢ* |

**Supplementary Table 5** Sequences of ChIP-qPCR primers used in this study

| **Position** | **Forward primer (5’-3’)** | **Reverse primer (5’-3’)** |
| --- | --- | --- |
| P1 (-752/-564) | CAGCAGTCATCCTACTCT | ATGGAGTTGTCGTGTAGA |
| P2 (-578/-347) | CACGACAACTCCATCATT | CAATCACTGAGGAAGCAA |
| P3 (-155/-80) | ACATTCTCCGTCTTCCAGG | TCTAGCTGTGGCTGGGAA |
| P4 (+334/+531) | TAATCTGCCGTCTGCCTA | CATCCACGCATCTCCTAA |

**Supplementary figures**

**
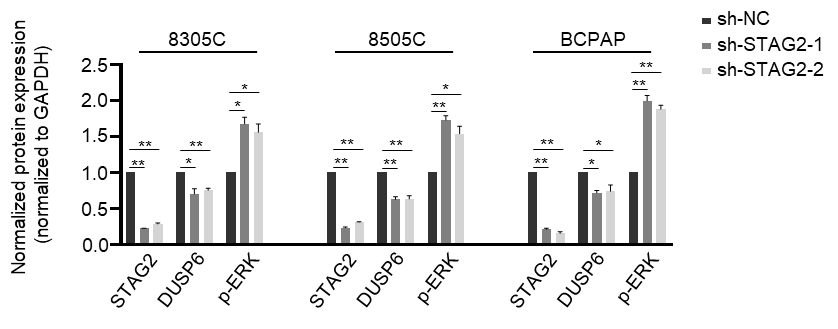
**

**Supplementary Fig. 1** Densitometric evaluation of relative protein expression normalized to GAPDH and statistical analysis of Figure 1d. Data are presented as mean ± SD of values. *, *P* <0.05; **, *P* <0.01.

**
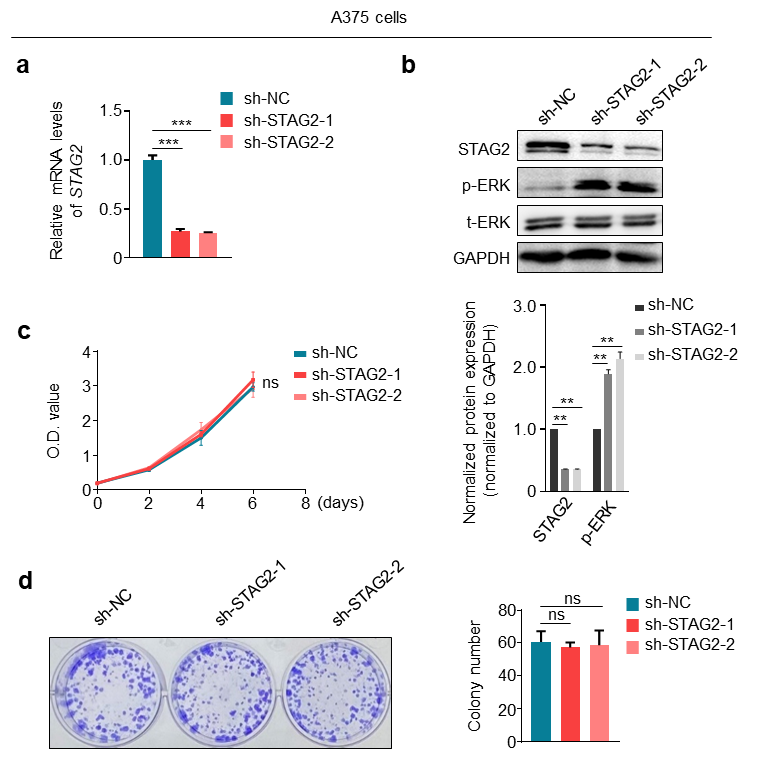
**

**Supplementary Fig. 2 The effect of STAG2 knockdown on the proliferation of melanoma cells.** **a** STAG2 knockdown efficiency in A375 cells was validated by qRT-PCR. β-actin was used as internal reference. **b** Western blot analysis of STAG2, p-ERK and t-ERK in STAG2-knockdown A375 cells and control cells (upper panel). Lower panel shows the densitometry ratio of the indicated proteins to GAPDH (loading control) and statistical analysis. The MTT (**c**) and colony formation (**d**) assays were used to evaluate the effect of STAG2 knockdown on cell proliferation and colony formation ability. For colony formation, left panel shows representative images of colony formation. Quantitative analysis of colony number was presented in the right panel. Data were presented as mean ± SD of values from three different experiments. ns, no significance; **, *P* <0.01; ***, *P* <0.001.

**
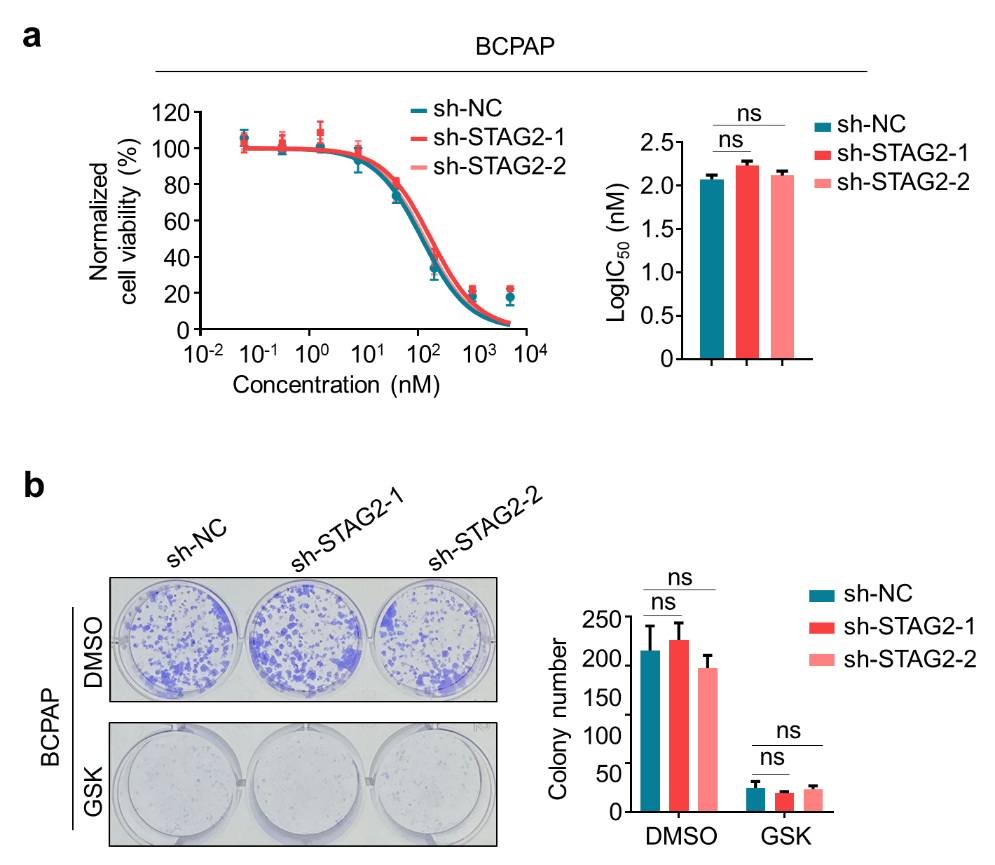
**

**Supplementary Fig. 3 The effect of STAG2 knockdown on** **the response of BCPAP cells to MEK inhibitor.** **a** STAG2-knockdown BCPAP and control cells were treated with different concentrations of MEK inhibitor GSK1120212 (GSK) for 72 h, and their effect on cell proliferation was assessed by MTT assay. The Reed-Muench method was used to calculate Log IC_50_ values. **b** Cells were treated with the 200 nM GSK or equivalent DMSO for 7-10 days, and then stained with crystal violet. The left panels show representative images of colony formation. Quantitative analysis of colony numbers is shown in the right panels. Data are presented as mean ± SD of values from three different experiments. ns, no significance.

**
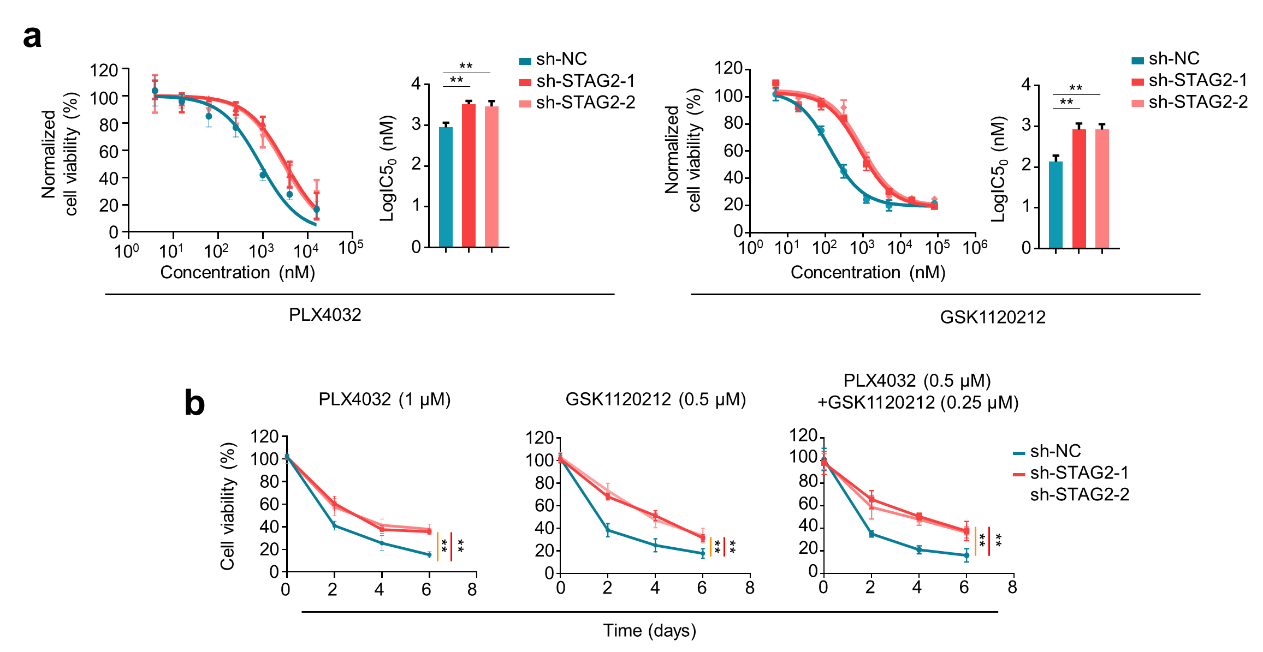
**

**Supplementary Fig. 4 The effect of STAG2 knockdown on drug sensitivity of melanoma cell.** **a** IC_50_ of BRAF^V600E^ inhibitor PLX4032 and MEK inhibitor GSK1120212 were calculated by MTT assay (left). LogIC50 values were presented as the mean ± SD (right). **b** Cells were treated with 1 μM PLX4032, 0.5 μM GSK1120212 or half of the above concentration for a combination treatment for the indicated time. The MTT assay was then performed to evaluate cell survival rate after drug treatment. Survival rate was calculated as the ratio of treatment group to non-treatment group at the same time. Data are presented as mean ± SD. **, *P* < 0.01.

**
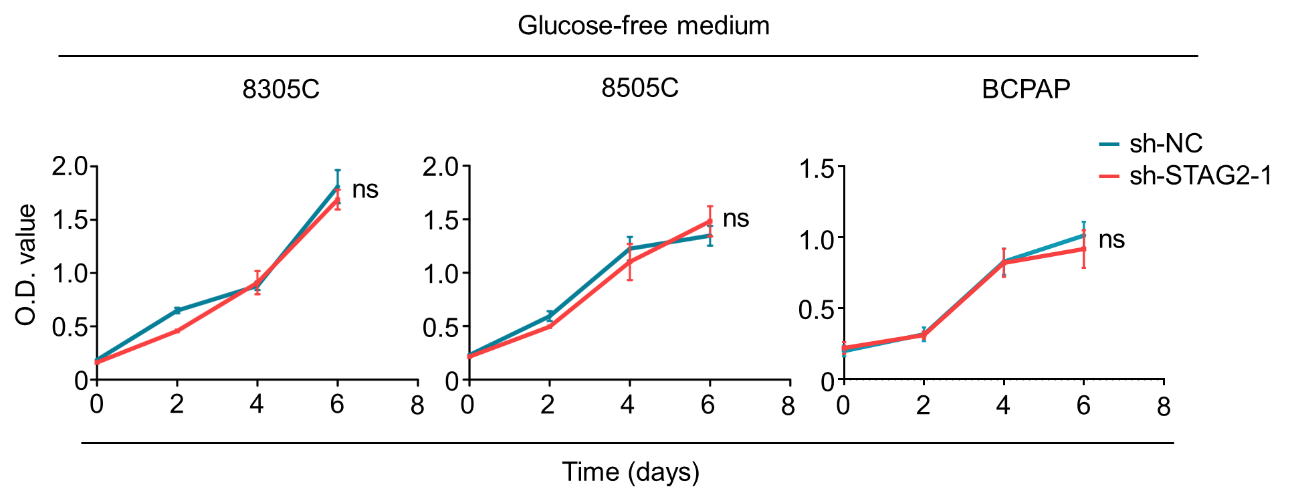
**

**Supplementary Fig. 5 The effect of STAG2 knockdown on the glucose dependence of thyroid cancer cells.** Glucose was deprived and MTT assay was then performed to evaluate the proliferation of STAG2-knockdown 8305C, 8505C and BCPAP cells and control cells. ns, no significance.

**Supplementary Fig. 6 Effect of STAG2 knockdown on the glutamine dependence of melanoma cells.** A375 cells were treated with different concentrations of glutamine, and MTT assay was then performed to evaluate cell viability. ns, no significance.

**Supplementary Fig. 7 Effect of STAG2 knockdown on thyroid cancer apoptosis upon glutamine deprivation.** 8505C and BCPAP cells were cultured in the medium containing normal glutamine (2 mM) or 50 nM glutamine (Gln-L) or were treated with 8 μM BPTES for 48 h. Cell apoptosis was then assessed by flow cytometry using Annexin V-FITC Detection Kit. Shown are the representative images of flow cytometry.

**Supplementary Fig. 8** Representative tumor sections were subjected to IHC staining with cleaved-PARP antibody and then quantitatively analyzed using AOD value. Scale bar, 200 μm. Data are presented as mean ± SD. ns, no significance; **, *P* <0.01.


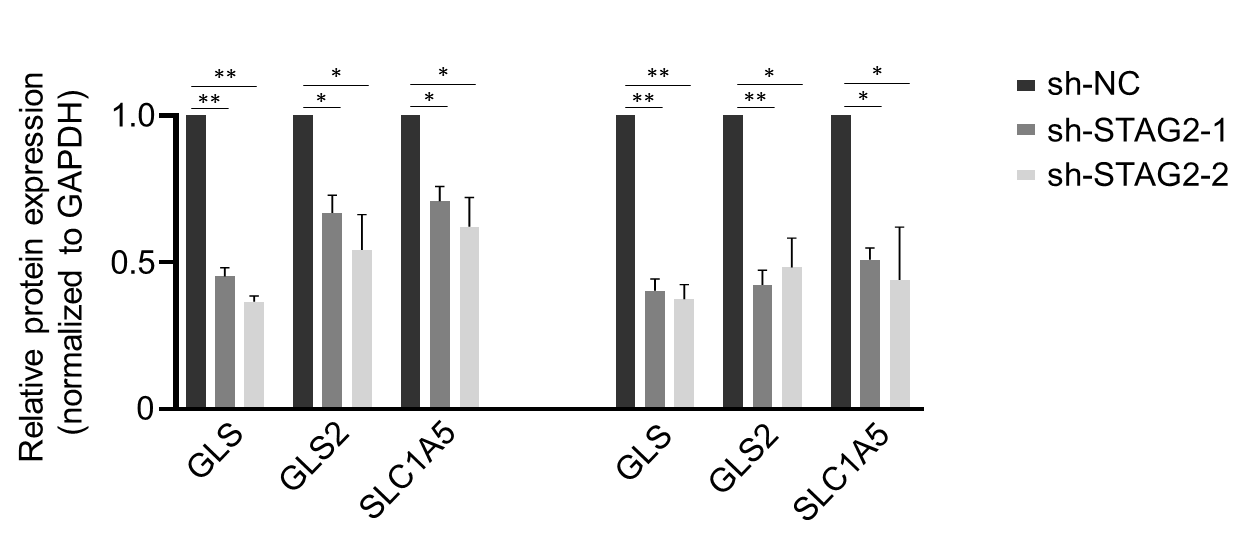


**Supplementary Fig. 9** Densitometric evaluation of relative protein expression normalized to GAPDH and statistical analysis in Figure 5b. Data are presented as mean ± SD of values. *, *P* <0.05; **, *P* <0.01.


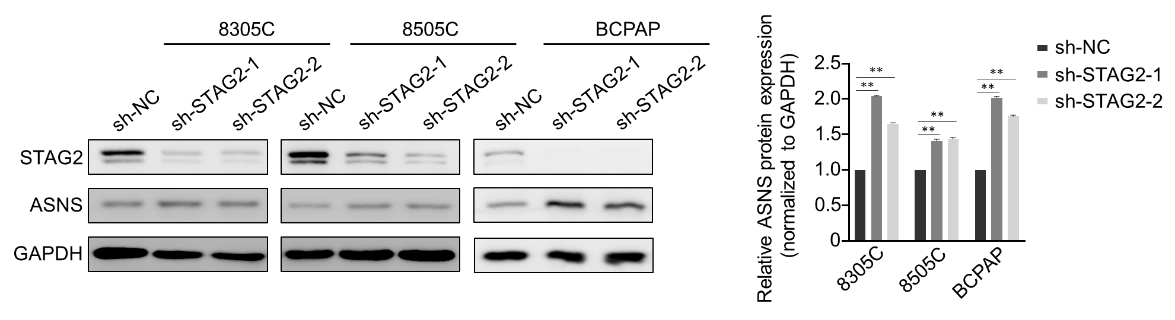


**Supplementary Fig. 10** The effect of STAG2 knockdown on protein expression of ASNS was analyzed by western blotting analysis (left panels). Densitometric evaluation of relative ASNS protein expression normalized to GAPDH (loading control) and statistical analysis was shown in the right panel. Data are presented as mean ± SD of values. **, *P* <0.01.

**Supplementary Fig. 11 Effect of STAG2 knockdown on GSH/GSSH ratio in thyroid cancer cells upon glutamine deprivation.** 8305C and 8505C cells were cultured in the medium containing 2 mM of glutamine (Control) or 50 nM of glutamine (Gln-L) or were treated with 8 μM BPTES for 24 h in the normal medium. Intracellular total GSH and GSSG were then measured using a GSH and GSSG Detection Assay Kit. Data are presented as mean ± SD of values. ns, no significance; *, *P* <0.05; **, *P* <0.01.


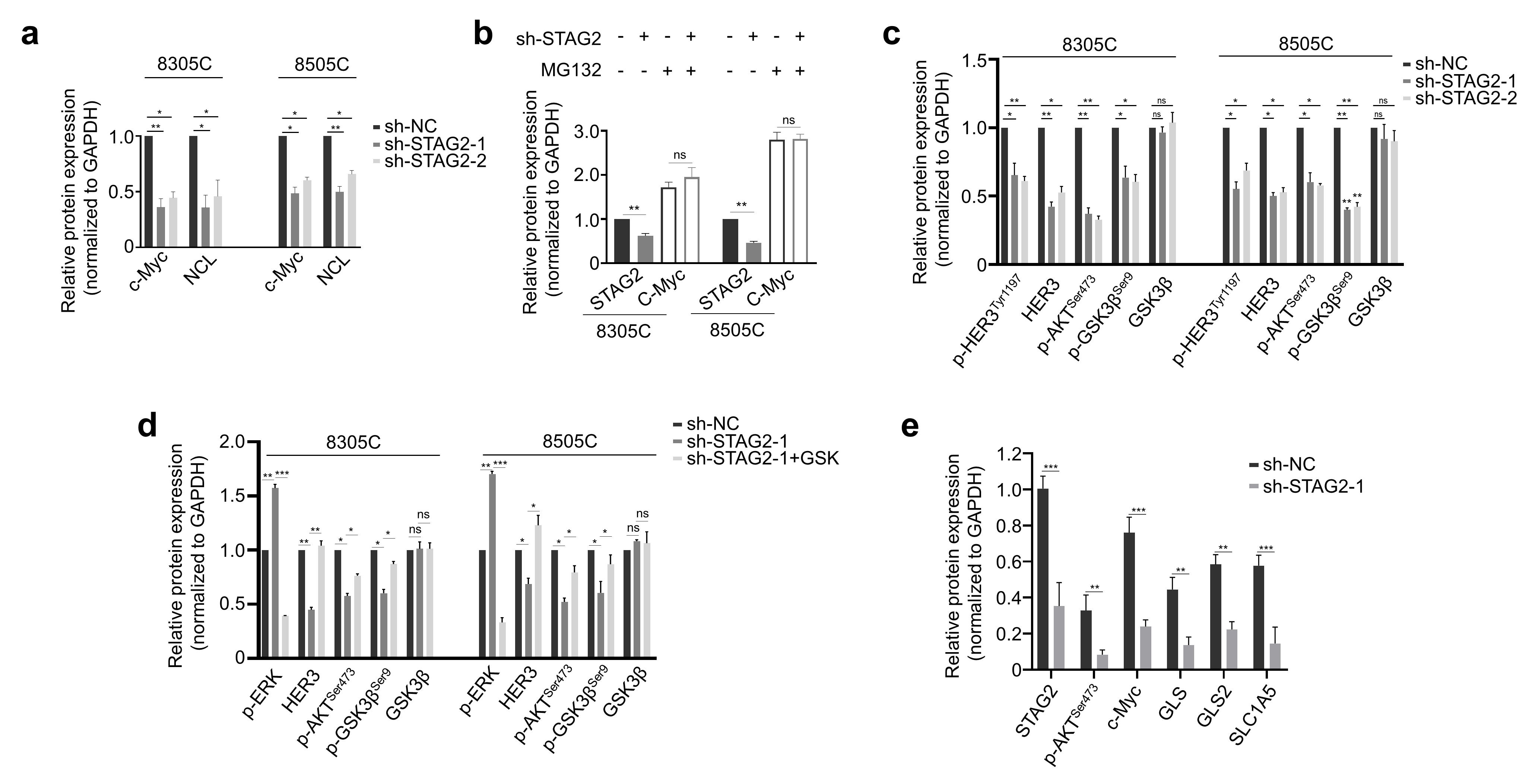


**Supplementary Fig. 12** Densitometric evaluation of relative protein expression normalized to GAPDH in Figures 6a (**a**), 6d (**b**), 6e (**c**), 6f (**d**) and 6g (**e**) and corresponding statistical analysis. Data are presented as mean ± SD of values. ns, no significance; *, *P* <0.05; **, *P* <0.01; ***, *P* <0.001.

**
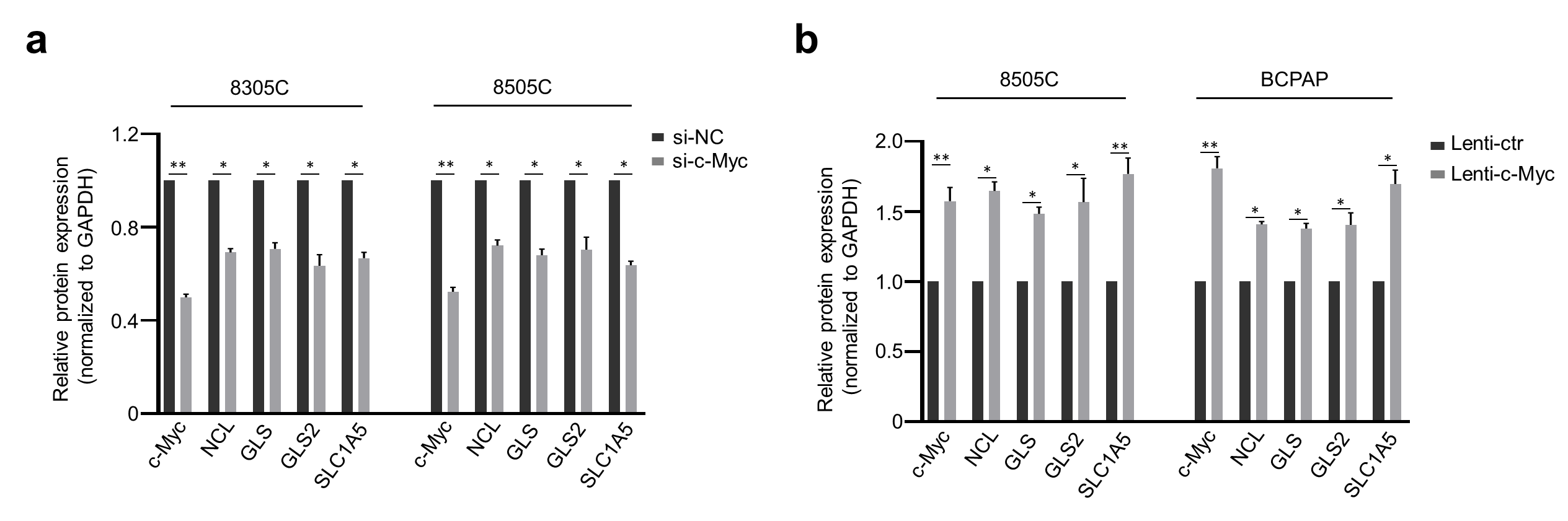
**

**Supplementary Fig. 13** Densitometric evaluation of relative protein expression normalized to GAPDH in Figure 7b (**a**) and 7d (**b**) and corresponding statistical analysis. Data are presented as mean ± SD of values. *, *P* <0.05; **, *P* <0.01.

**
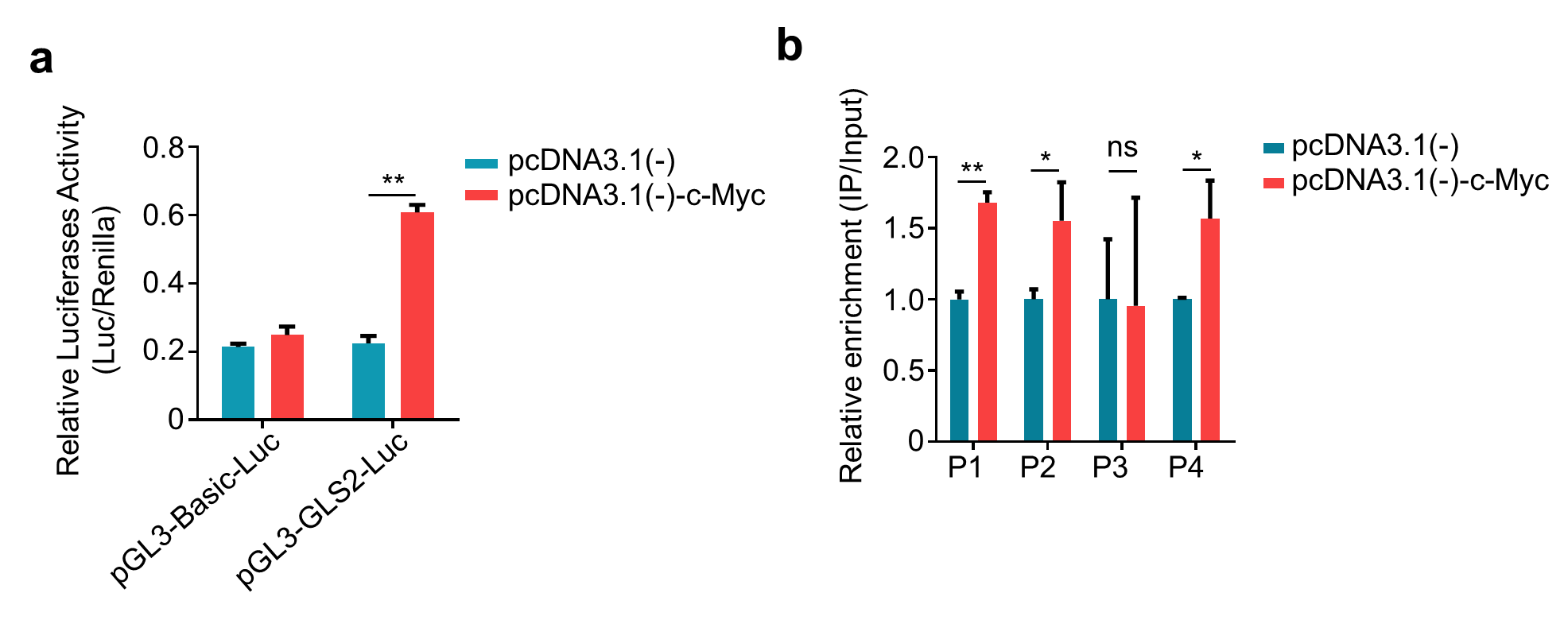
**

**Supplementary Fig. 14 Identification of GLS2 as downstream targets of c-Myc. a** 293T cells ectopically expressing c-Myc and control cells were then transfected with pGL3-Basic or luciferase reporter construct containing the promoter region of GLS2 (-856/+732) genes (pGL3-GLS2-Luc). Co-transfection with empty vector was used as control. The Luc/Renilla activity ratio was shown as mean ± SD of three independent assays. **b** 293T cell transfected with c-Myc or controlled plasmid was subjected to ChIP-qPCR assays using anti-c-Myc antibody. Fold enrichment of the four selected regions was shown as mean ± SD of three independent assays. ns, no significance; *, *P* <0.05; **, *P* <0.01.


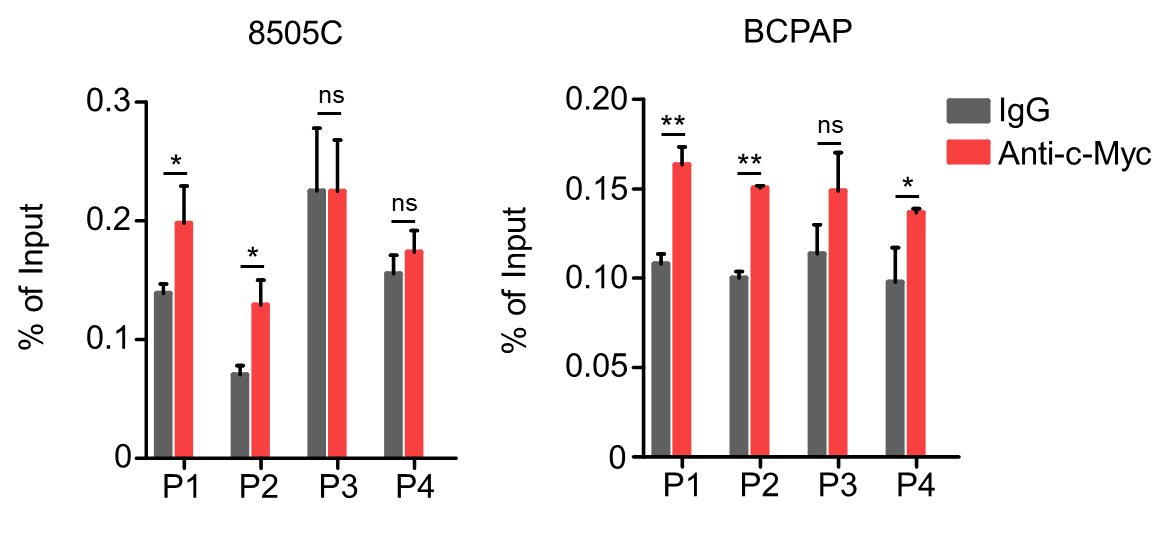


**Supplementary Fig. 15 Identification of GLS2 as downstream targets of c-Myc.** 8505C and BCPAP cells were collected, lysed and subjected to ChIP-qPCR assays using anti-c-Myc or IgG antibodies. Four regions (P1: -752/-564; P2: -578/-347; P3: -155/-80; P4: +334/+531) within GLS2 promoter were selected for ChIP-qPCR assays, respectively. Fold enrichment was shown as mean ± SD of three independent assays. ns, no significance; *, *P* <0.05; **, *P* <0.01.

**Supplementary Fig. 16 The effect of ectopic expression of c-Myc on the proliferation and colony formation ability of thyroid cancer cells in different conditions. a** MTT assay was performed to assess the proliferation ability of BCPAP cells with the indicated treatments. **b** Colony formation ability of BCPAP cells with the indicated treatments: Control (2 mM of glutamine), Gln-L (50 nM of glutamine) and BPTES (8 μM BPTES in the normal medium). The left panels show representative images and the colony number is quantified in the right panel. Data are presented as mean ± SD of values. ns, no significance; *, *P* <0.05; **, *P* <0.01; ***, *P* <0.001.

**Supplementary Fig. 17 The effect of ectopic expression of c-Myc on thyroid cancer cell apoptosis in different conditions. a** 8505C cells were subjected to the indicated treatments for 48 h, and apoptotic cells were then detected by flow cytometry using Annexin V-FITC Detection Kit. **b** The percentage of apoptotic cells calculated as the sum of the early and late apoptosis was presented. Data are presented as mean ± SD. ns, no significance; **, *P* <0.01; ***, *P* <0.001.

**
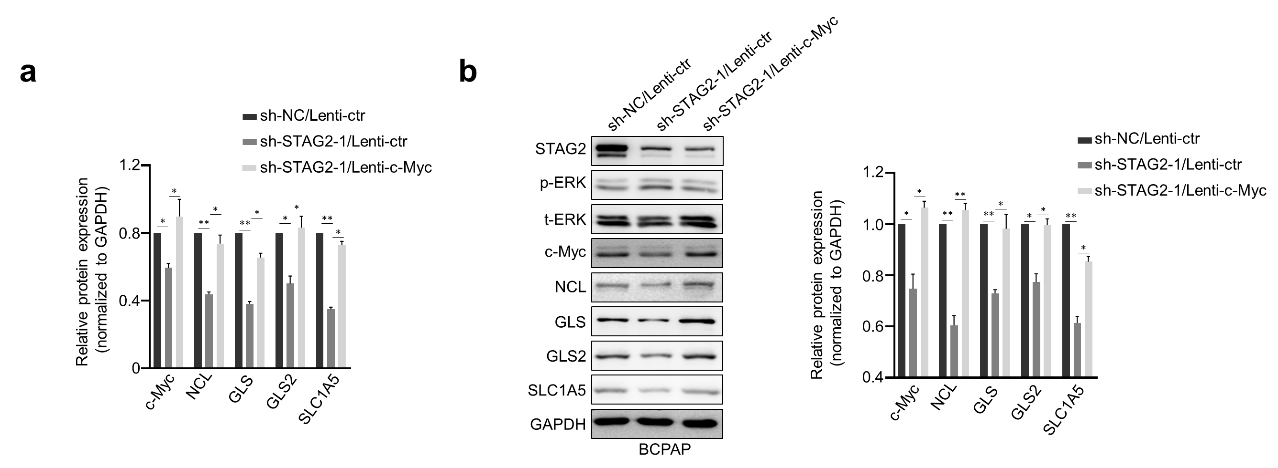
**

**Supplementary Fig. 18** **STAG2 affects the glutamine dependence of thyroid cancer cells via c-Myc.** **a** Densitometric evaluation of relative protein expression normalized to GAPDH in Figure 7g and corresponding statistical analysis. **b** Left panel shows western blot analysis of the indicated proteins in BCPAP cells. The right panel shows the densitometric evaluation of relative protein expression normalized to GAPDH and corresponding statistical analysis. Data are presented as mean ± SD of values. *, *P* <0.05; **, *P* <0.01.
