# Supplementary material for: STAG2 inactivation reprograms glutamine metabolism of BRAF-mutant thyroid cancer cells
Source: Cell Death Dis. 2023 Jul 21;14(7):454. doi: 10.1038/s41419-023-05981-z (PMC10361981; doi:10.1038/s41419-023-05981-z)

Fig. 1d

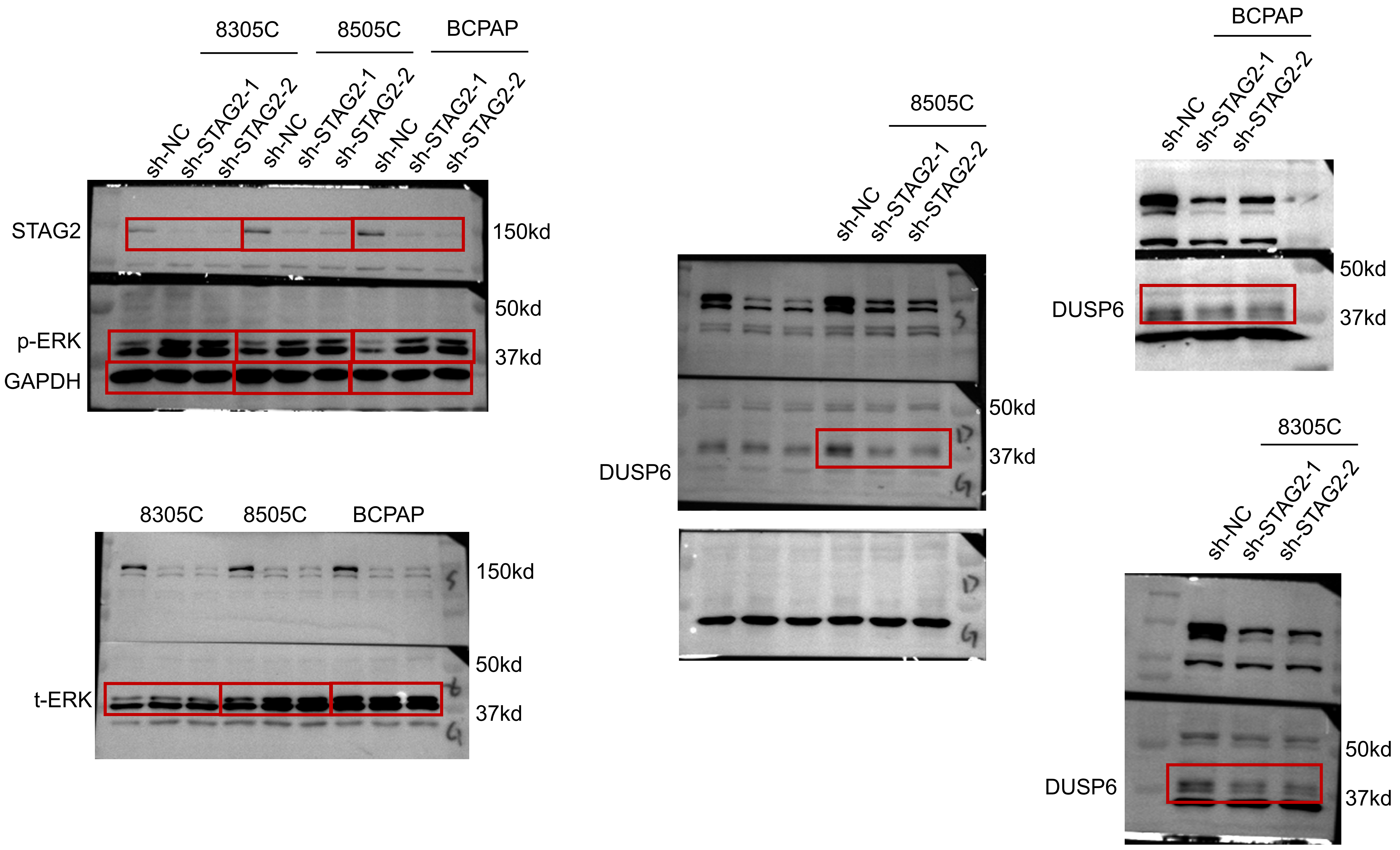

8305C
8505C
BCPAP
sh-NC
sh-STAG2-1
sh-STAG2-2
STAG2
p-ERK
GAPDH
150kd
50kd
37kd
t-ERK
DUSP6

Fig. 5b

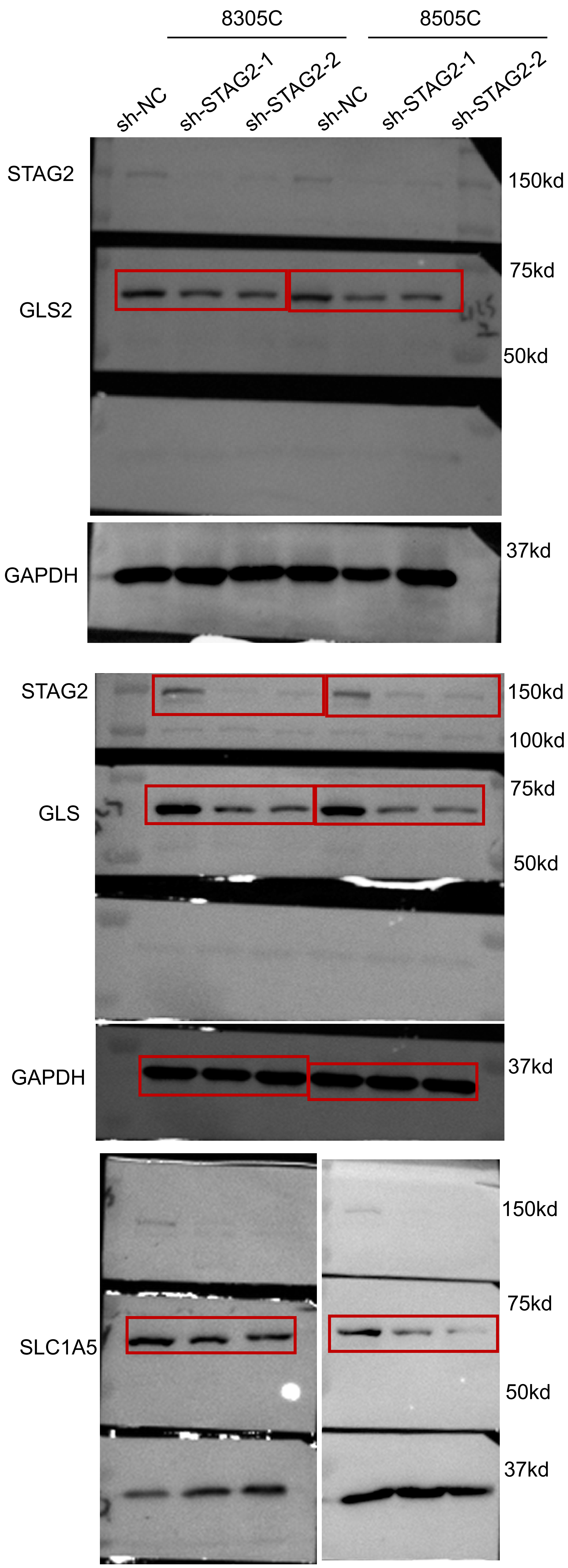
8305C
8505C
sh-NC
sh-STAG2-1
sh-STAG2-2
sh-NC
sh-STAG2-1
sh-STAG2-2
STAG2
150kd
GLS2
75kd
50kd
GAPDH
37kd
STAG2
150kd
100kd
GLS
75kd
50kd
GAPDH
37kd
SLC1A5
150kd
75kd
50kd
37kd

Fig. 6a

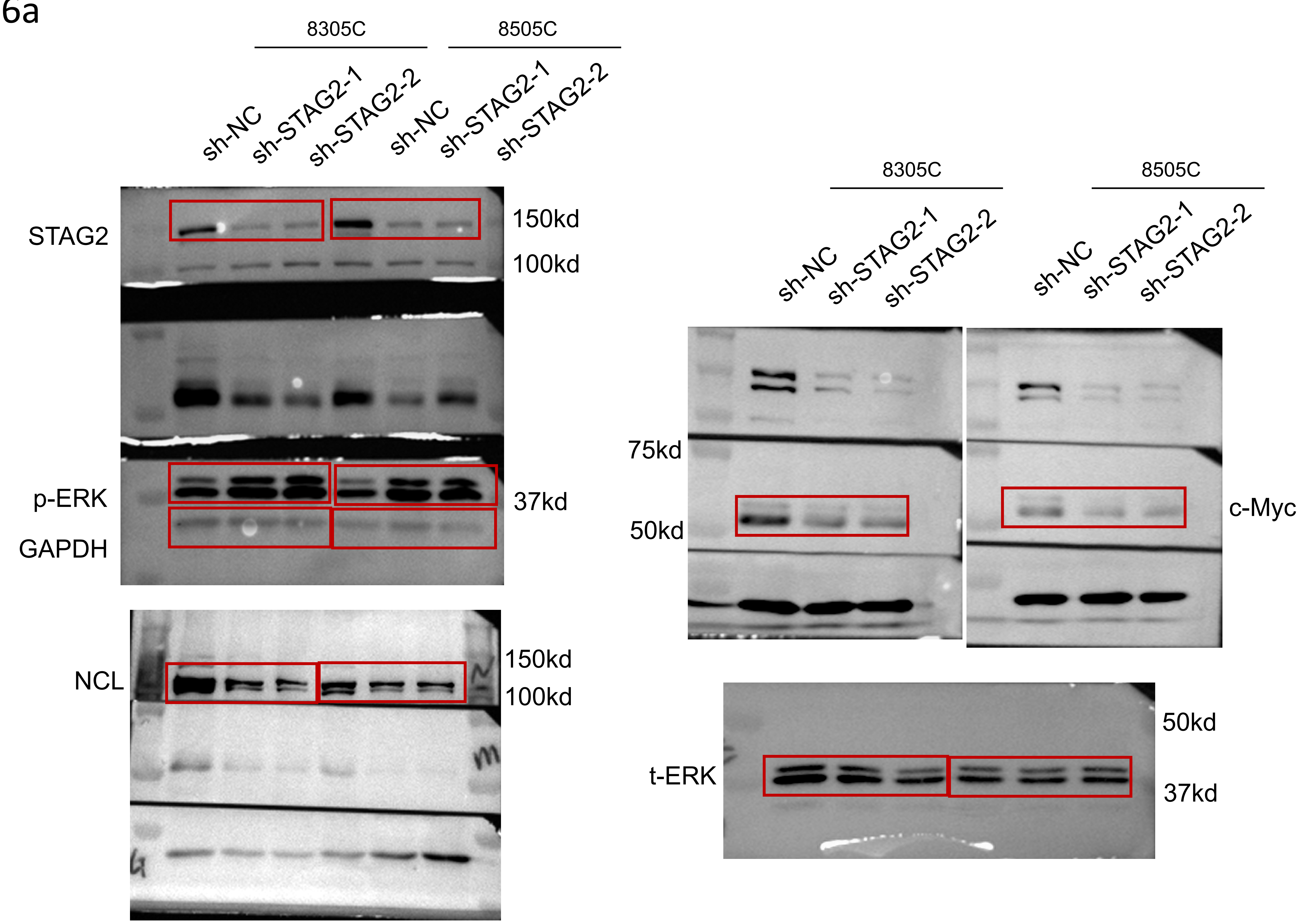
8305C
8505C
sh-NC
sh-STAG2-1
sh-STAG2-2
sh-NC
sh-STAG2-1
sh-STAG2-2
STAG2
150kd
100kd
p-ERK
GAPDH
37kd
NCL
150kd
100kd
8305C
8505C
sh-NC
sh-STAG2-1
sh-STAG2-2
sh-NC
sh-STAG2-1
sh-STAG2-2
75kd
50kd
c-Myc
t-ERK
50kd
37kd

Fig. 6c

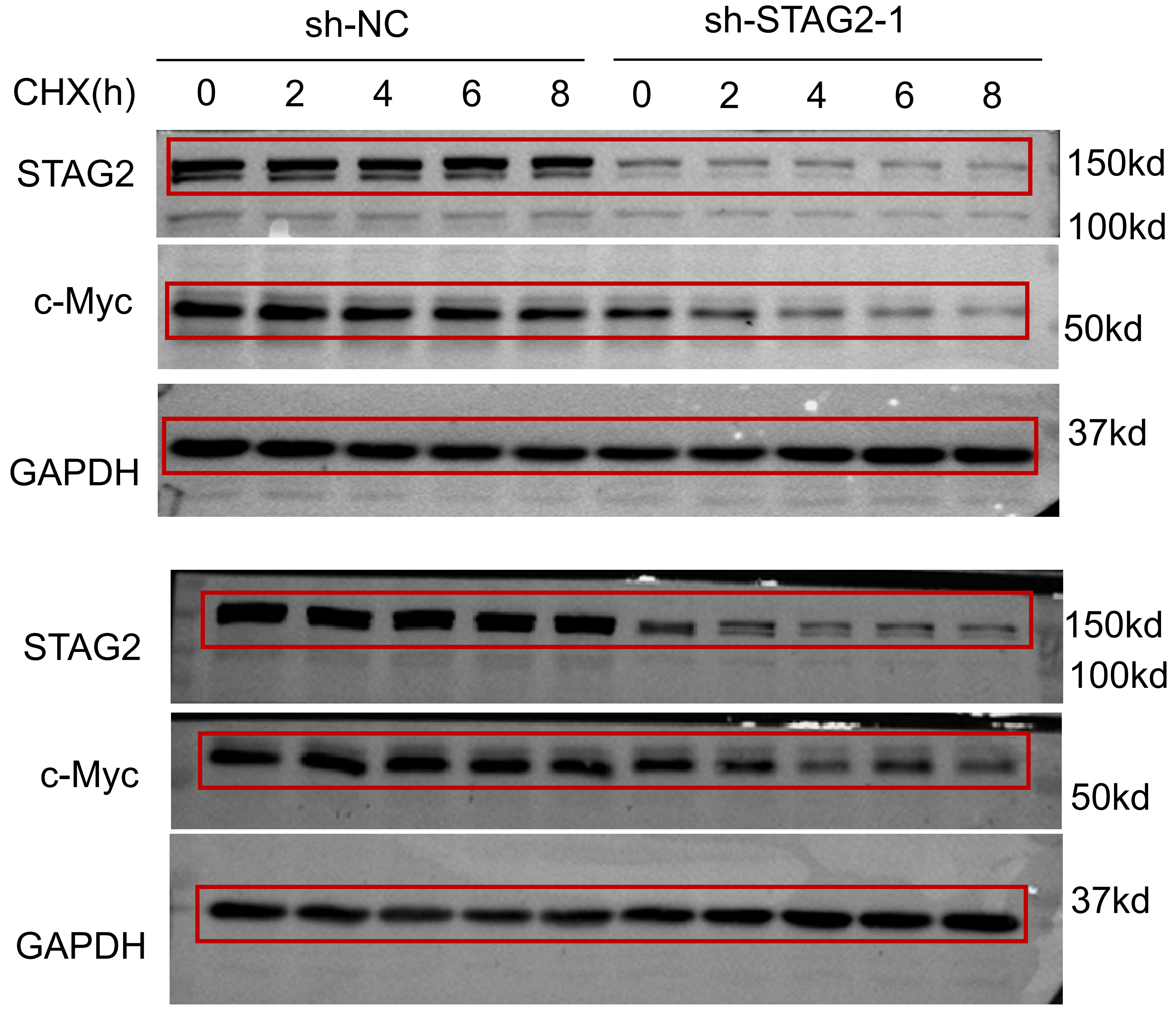
sh-NC
sh-STAG2-1
CHX(h)
0
2
4
6
8
0
2
4
6
8
STAG2
150kd
100kd
c-Myc
50kd
GAPDH
37kd
STAG2
150kd
100kd
c-Myc
50kd
GAPDH
37kd

Fig. 6d

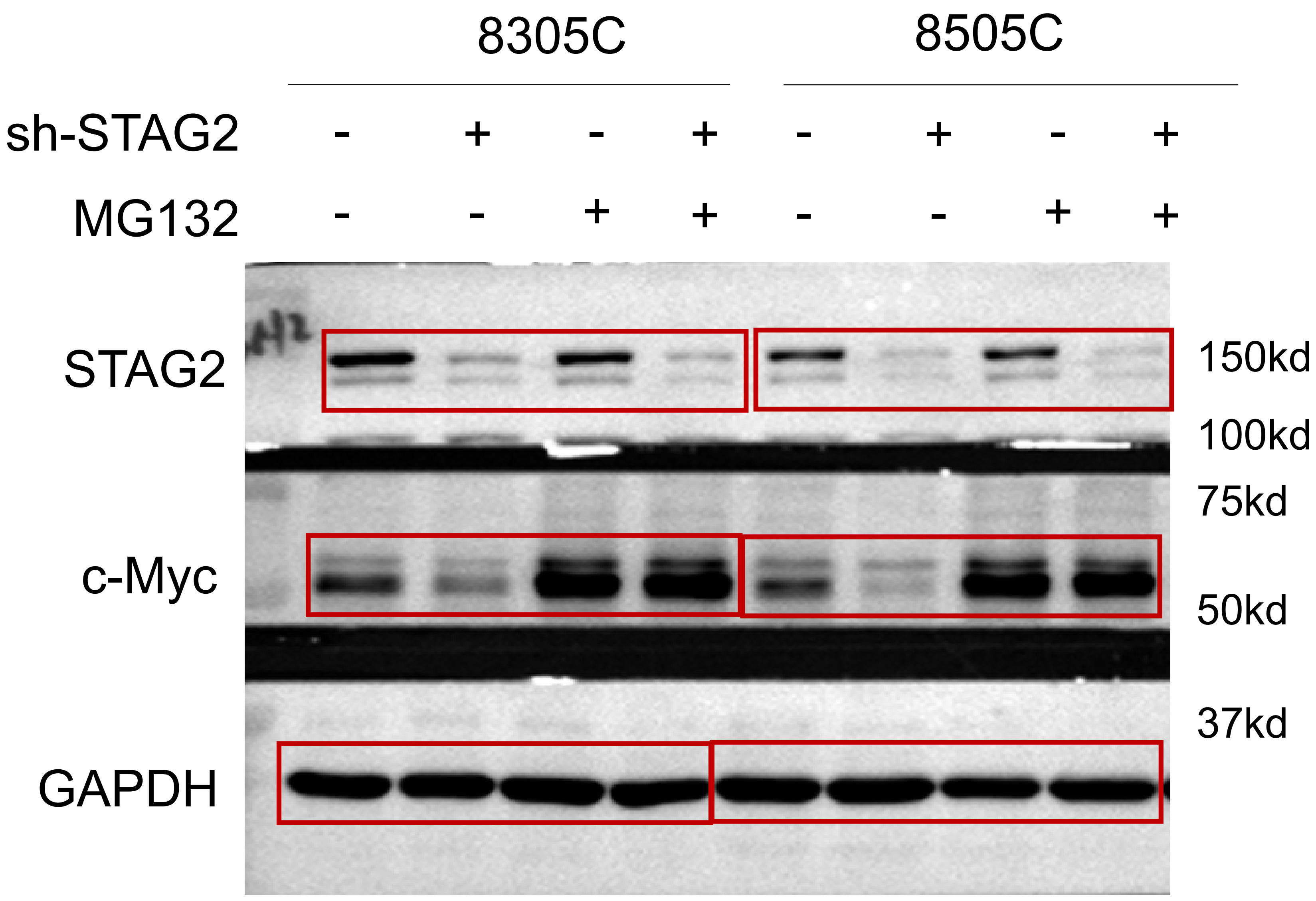
8305C
8505C
sh-STAG2 - + - + - + - +
MG132 - - + + - - + +
STAG2
c-Myc
GAPDH
150kd
100kd
75kd
50kd
37kd

Fig.6e

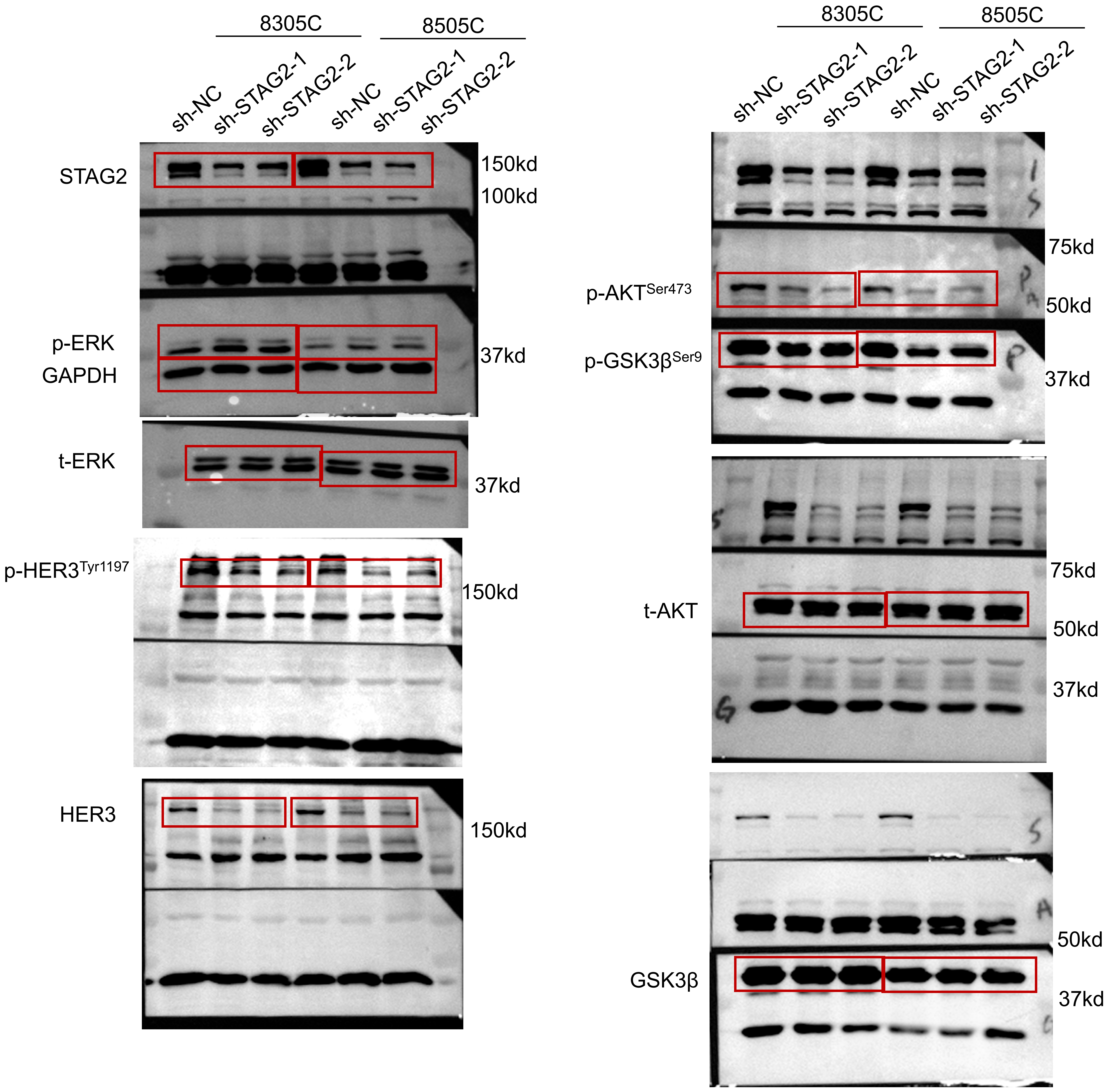

8305C
8505C
sh-NC
sh-STAG2-1
sh-STAG2-2
sh-NC
sh-STAG2-1
sh-STAG2-2
STAG2
150kd
100kd
p-ERK
GAPDH
37kd
t-ERK
37kd
p-HER3Tyr1197
150kd
HER3
150kd
8305C
8505C
sh-NC
sh-STAG2-1
sh-STAG2-2
sh-NC
sh-STAG2-1
sh-STAG2-2
75kd
p-AKTSer473
50kd
p-GSK3βSer9
37kd
75kd
t-AKT
50kd
37kd
50kd
GSK3β
37kd

Fig. 6f

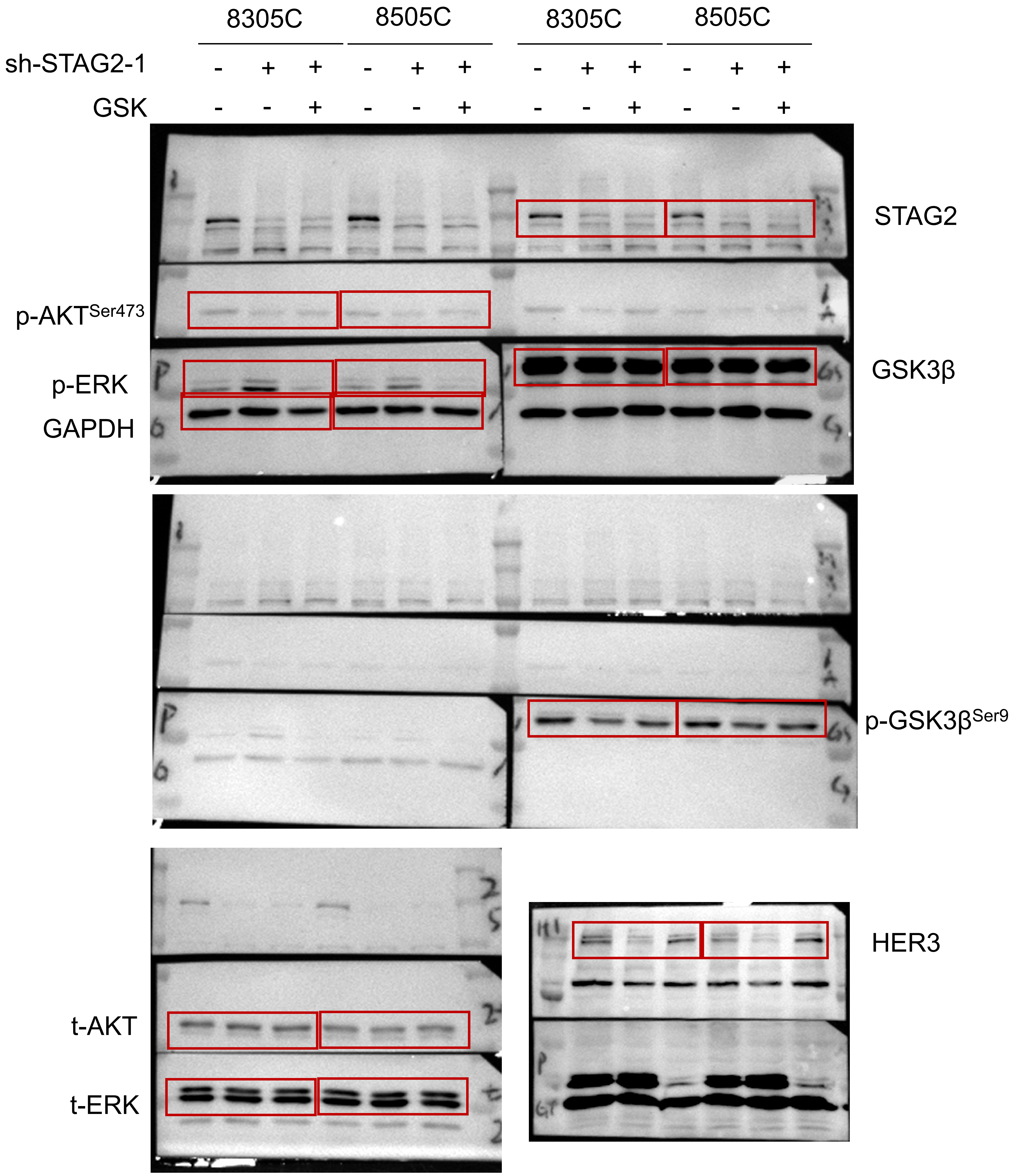

8305C
8505C
8305C
8505C
sh-STAG2-1
- + + - + + - + + - + +
GSK
- - + - - + - - + - - +
STAG2
p-AKT^Ser473
p-ERK
GAPDH
GSK3β
p-GSK3β^Ser9
t-AKT
t-ERK
HER3

Fig. 6g

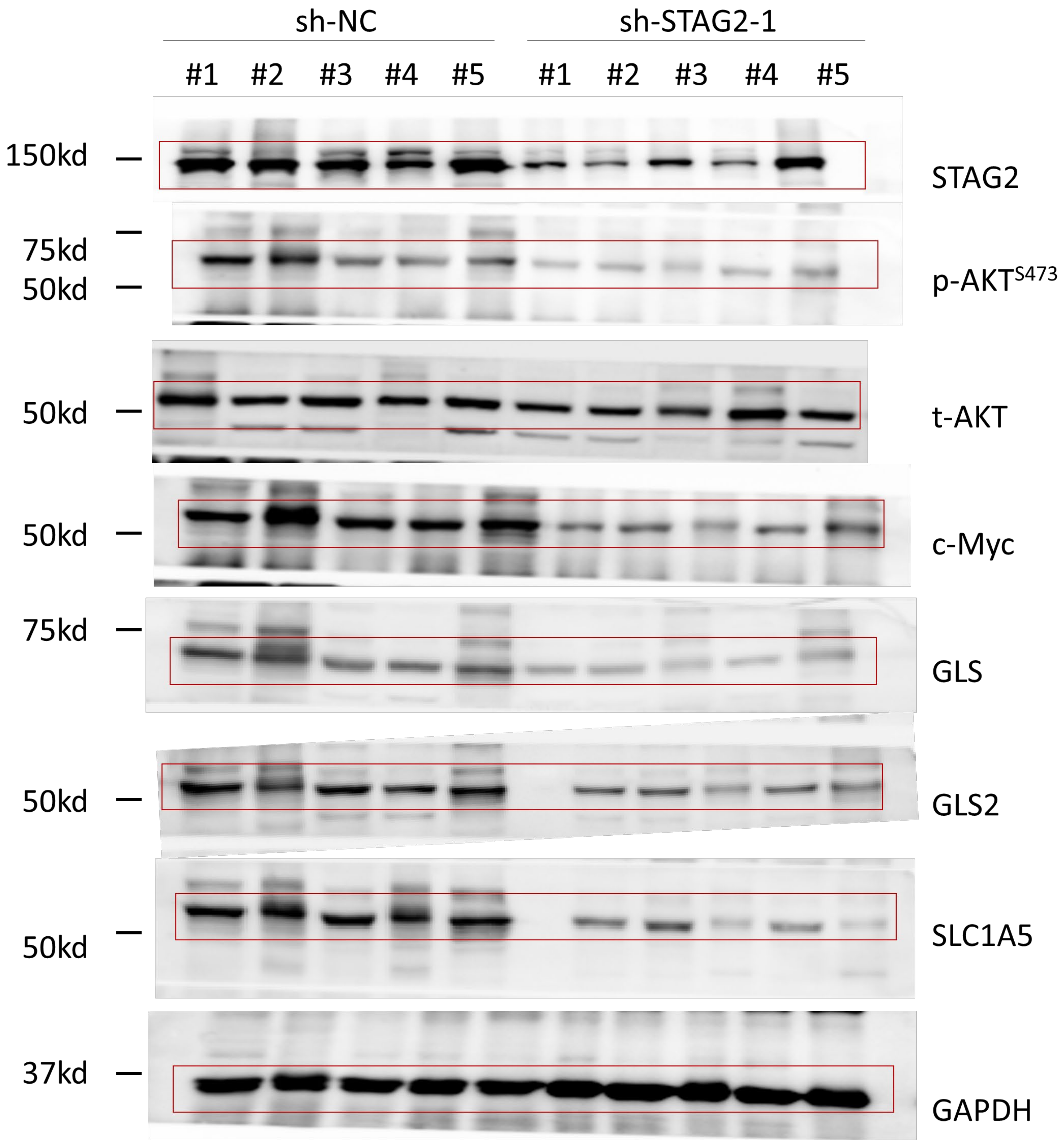

sh-NC
sh-STAG2-1
#1
#2
#3
#4
#5
#1
#2
#3
#4
#5
150kd
75kd
50kd
50kd
50kd
75kd
50kd
50kd
37kd
STAG2
p-AKT S473
t-AKT
c-Myc
GLS
GLS2
SLC1A5
GAPDH

Fig. 7b

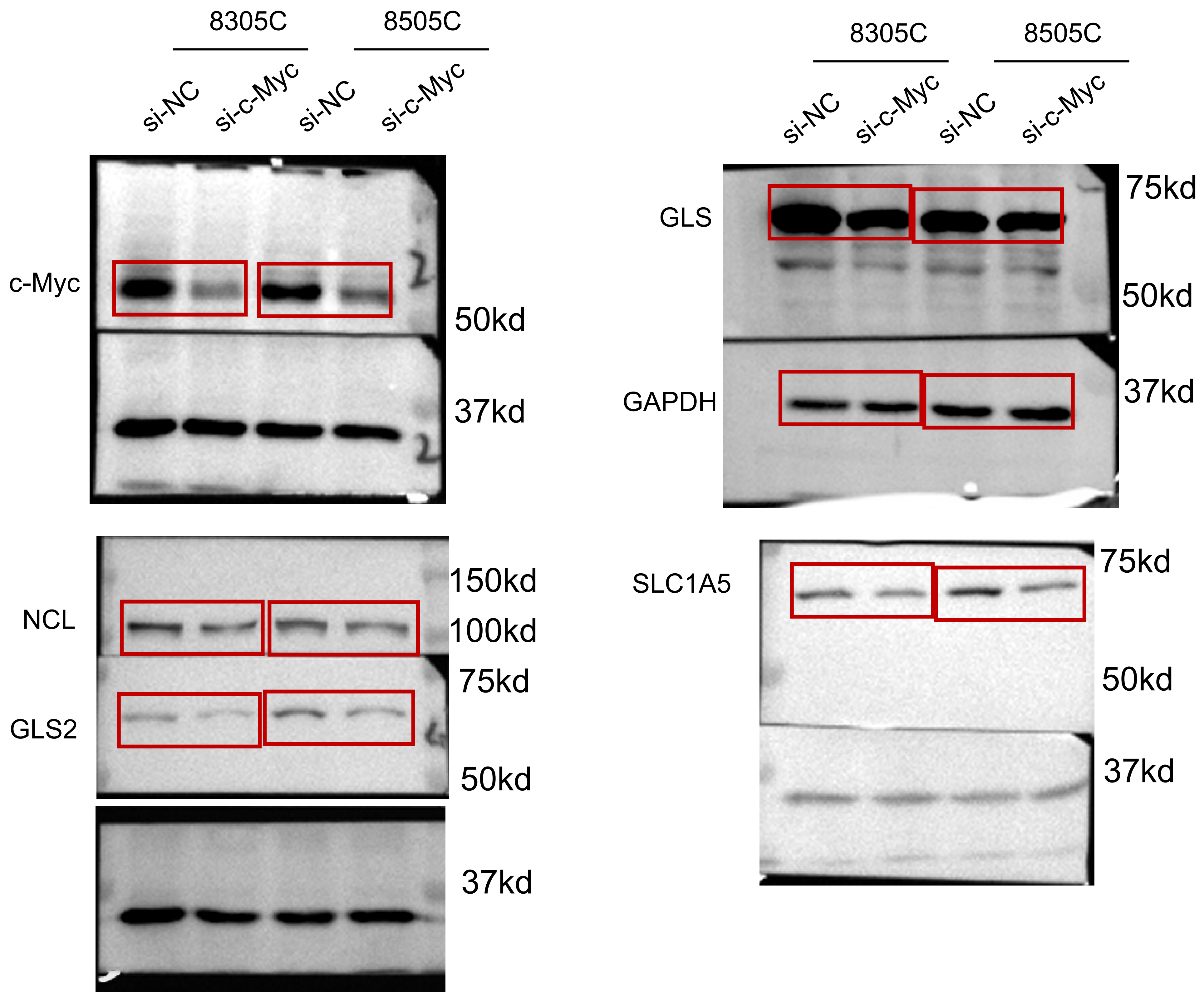

8305C
8505C
si-NC
si-c-Myc
si-NC
si-c-Myc
c-Myc
50kd
37kd
NCL
GLS2
150kd
100kd
75kd
50kd
37kd
GLS
GAPDH
75kd
50kd
37kd
SLC1A5
75kd
50kd
37kd

Fig. 7d

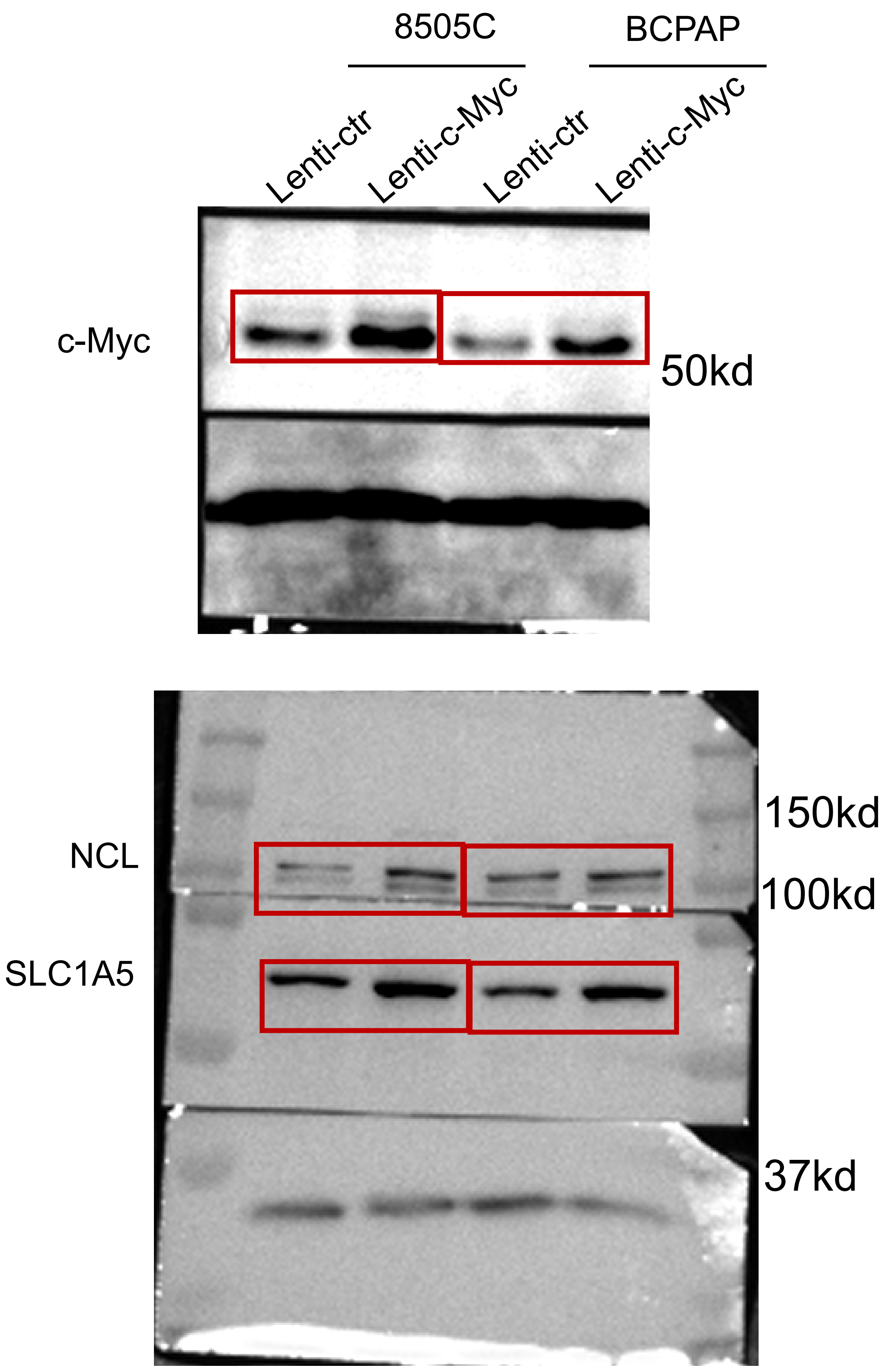

8505C
BCPAP
Lenti-ctr
Lenti-c-Myc
Lenti-ctr
Lenti-c-Myc
c-Myc
50kd
NCL
SLC1A5
150kd
100kd
37kd

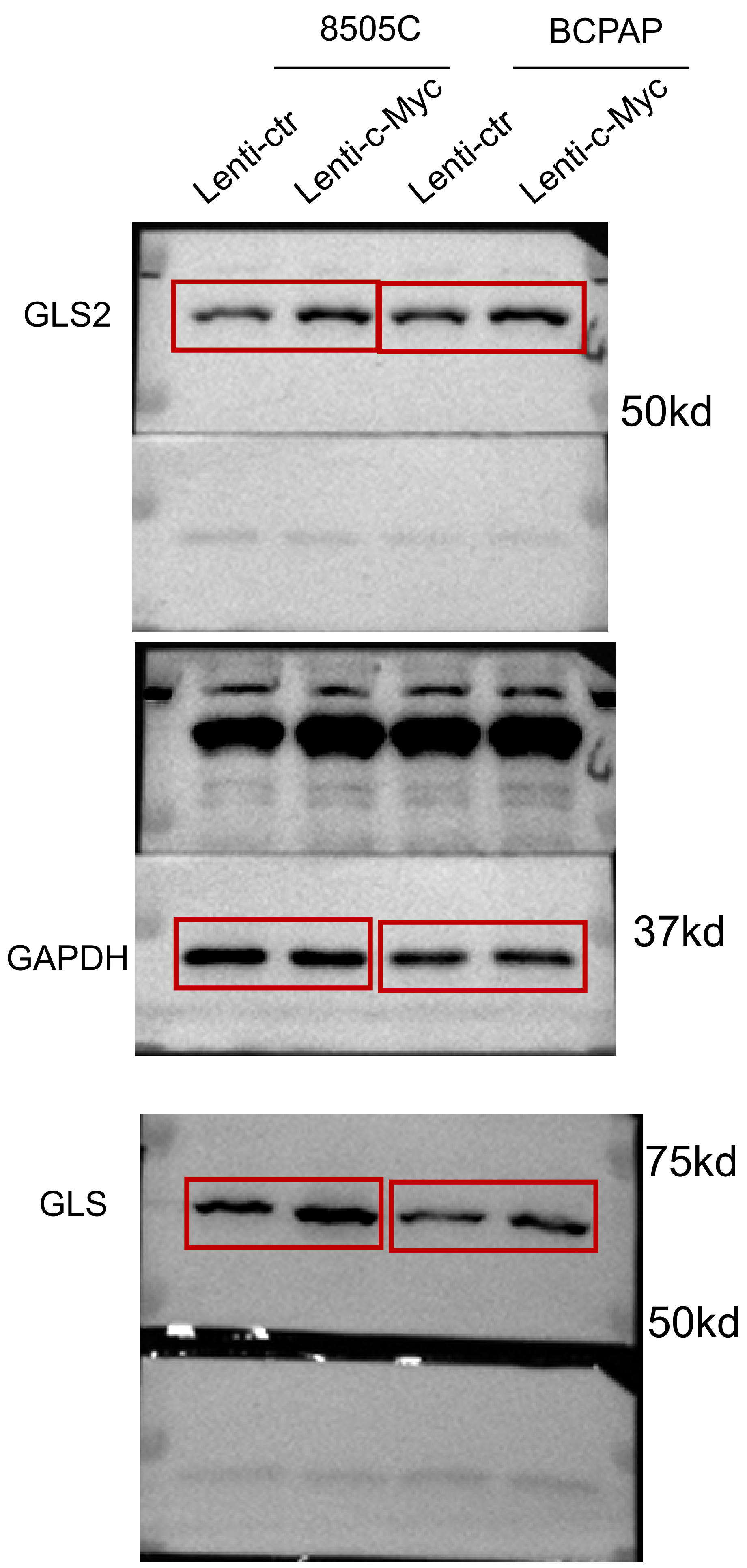

8505C
BCPAP
Lenti-ctr
Lenti-c-Myc
Lenti-ctr
Lenti-c-Myc
GLS2
50kd
GAPDH
37kd
GLS
75kd
50kd

Fig. 7g and Supplementary Fig. 18b

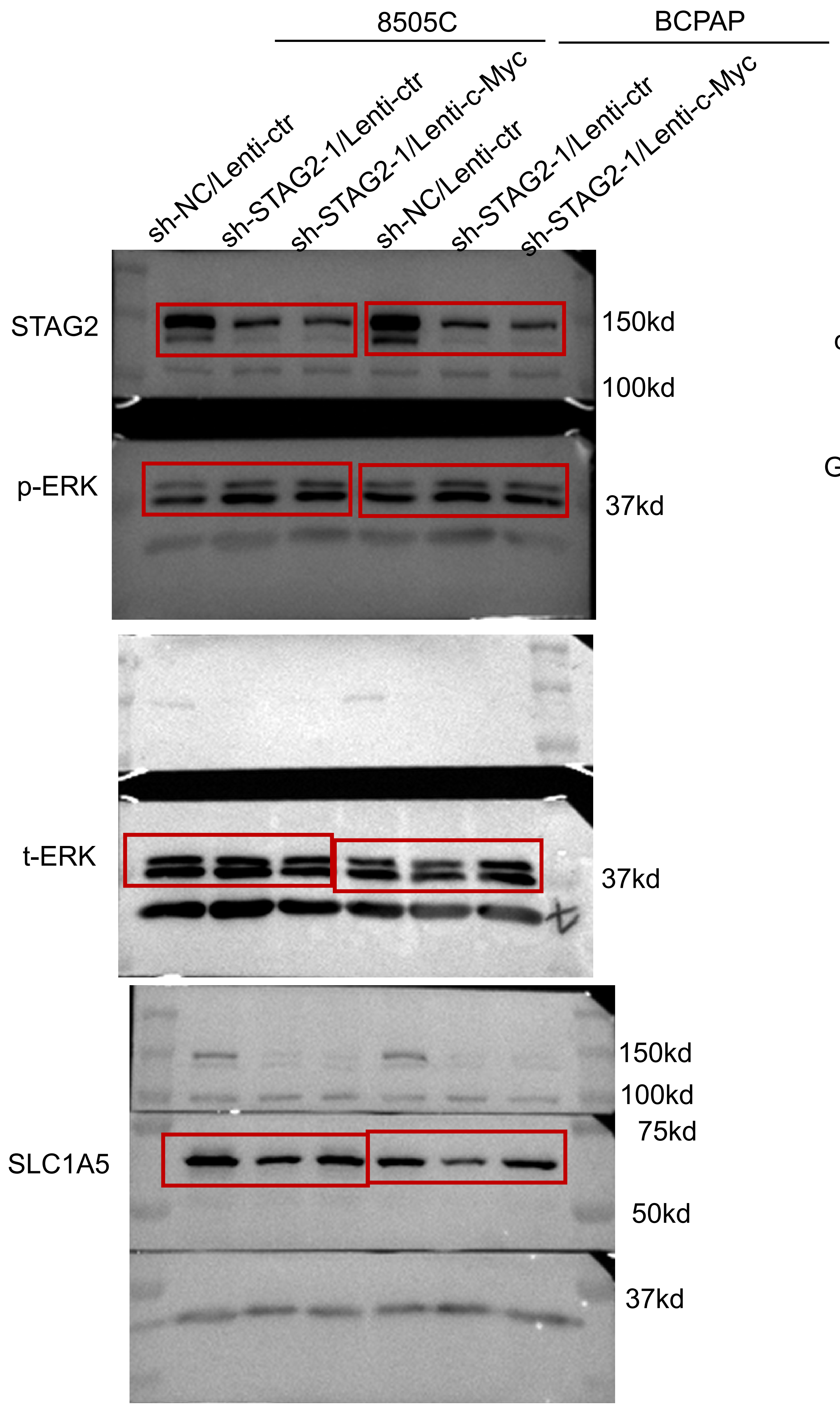

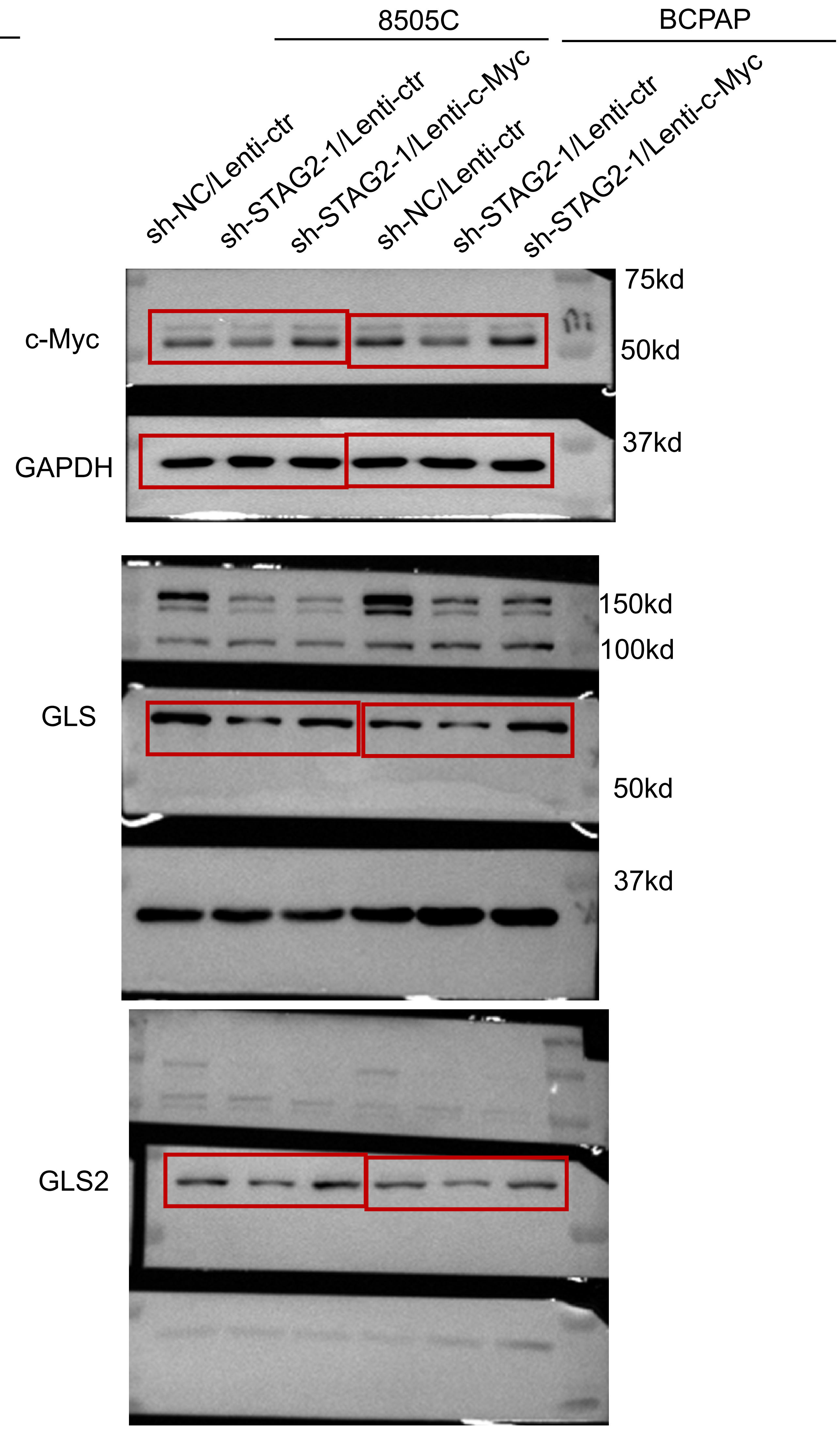

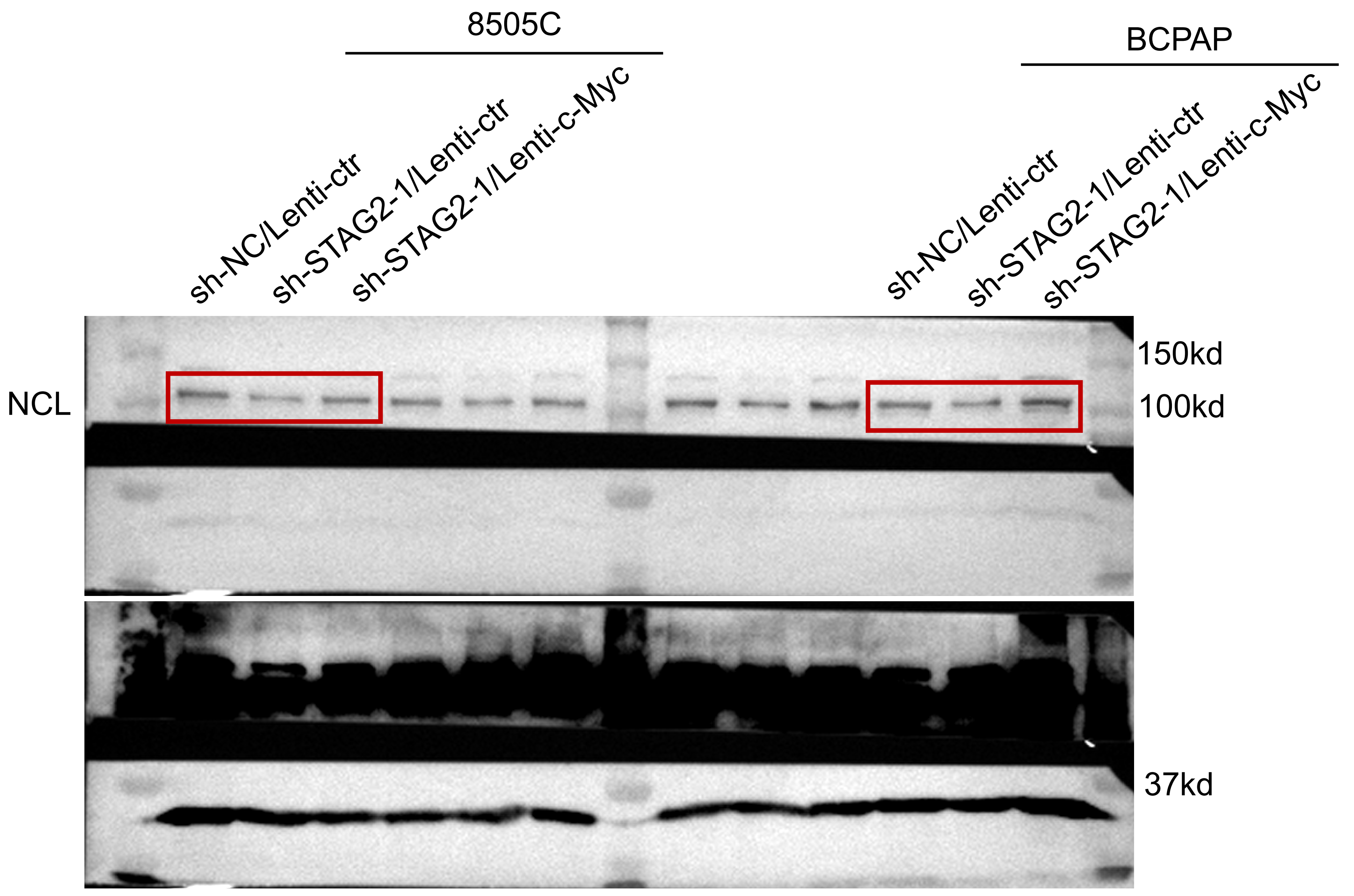

Supplementary Fig. 2b

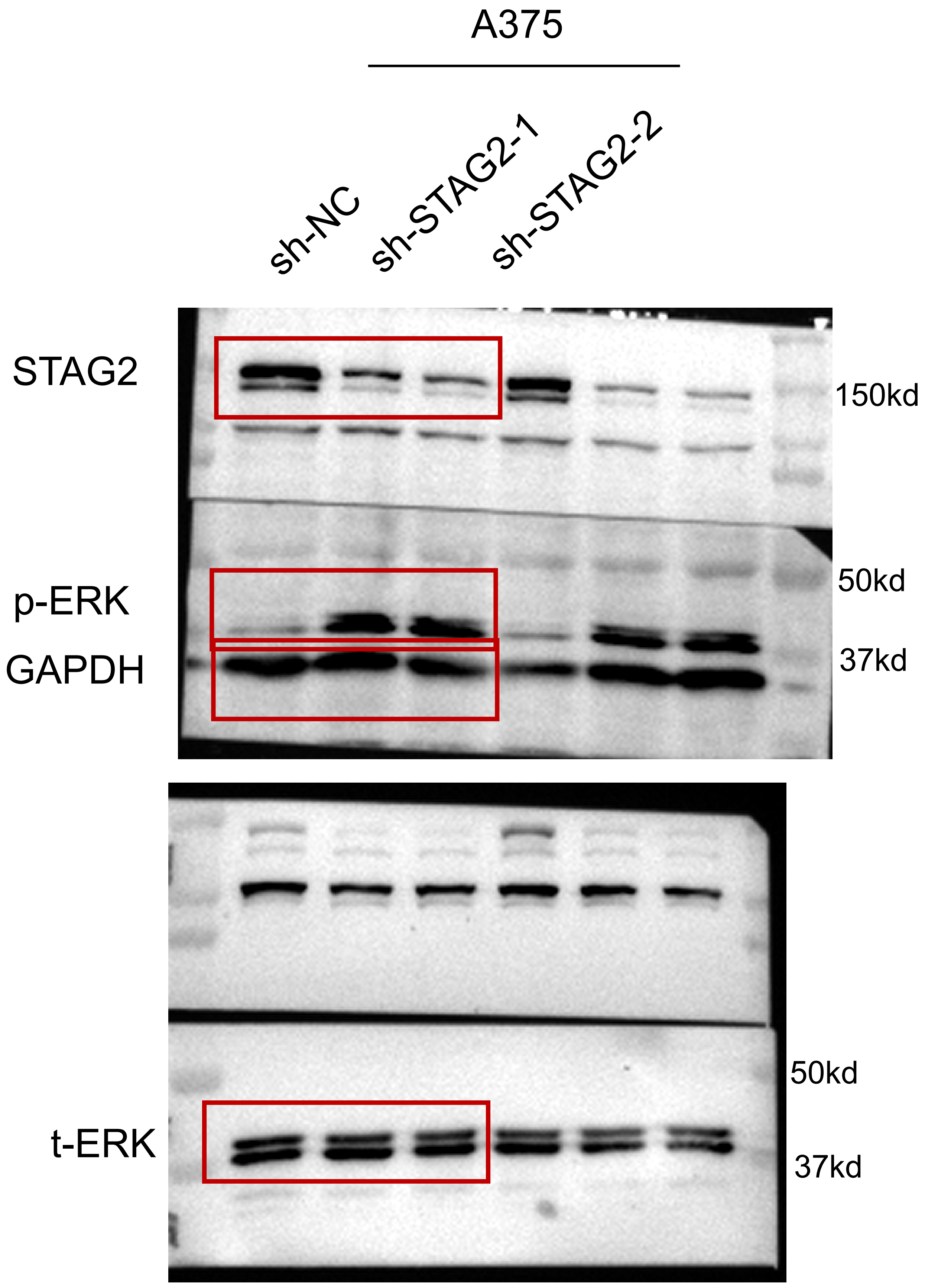

Supplementary Fig. 10

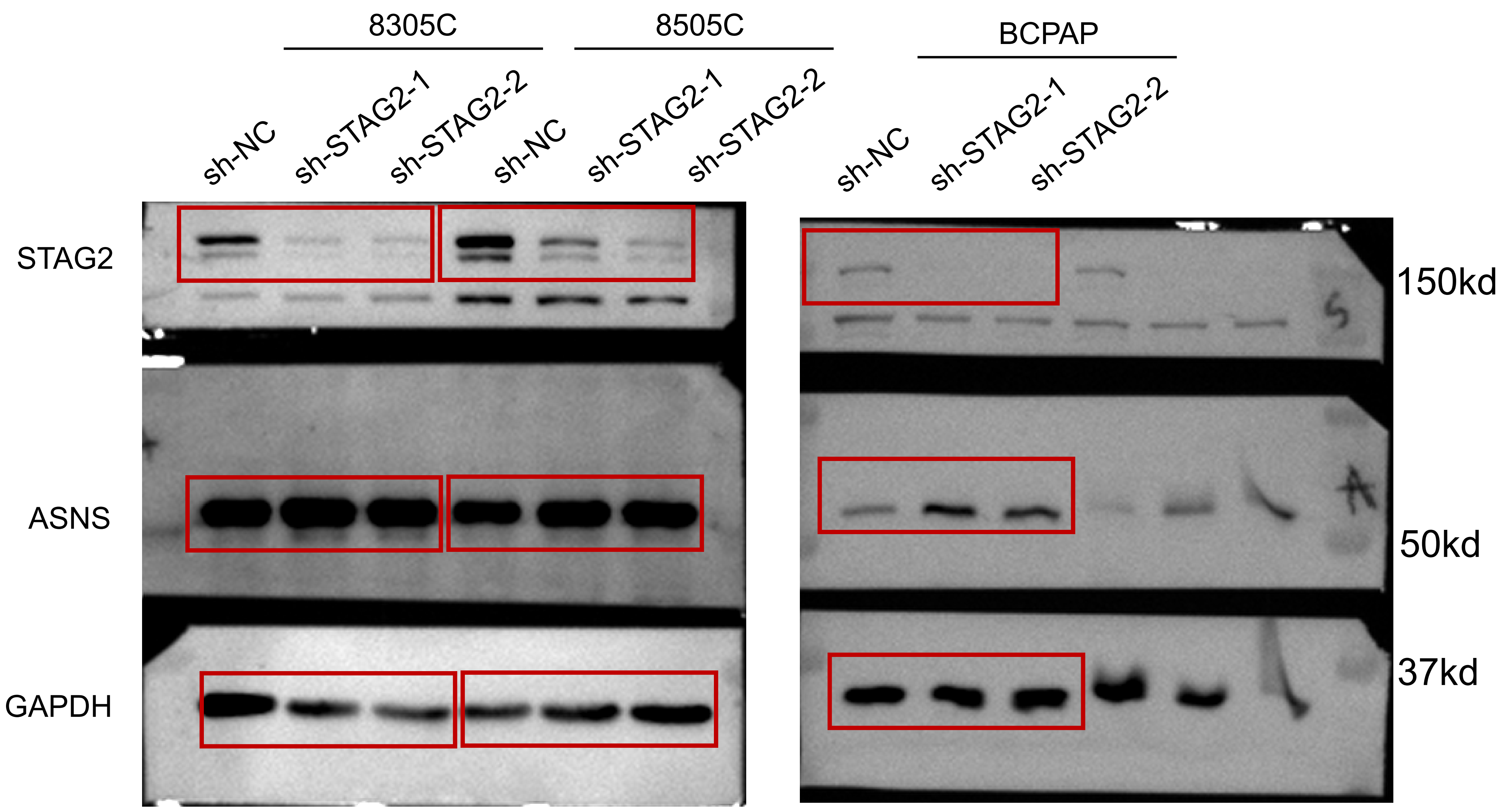

Supplement: Supplementary file 2 — Original Data File [file 41419_2023_5981_MOESM2_ESM.pdf]
